# Supplementary material for: AntimiR targeting of microRNA-134 reduces seizures in a mouse model of Angelman syndrome
Source: Mol Ther Nucleic Acids. 2022 Apr 20;28:514–29. doi: 10.1016/j.omtn.2022.04.009 (PMC9092865; doi:10.1016/j.omtn.2022.04.009)
Supplement: Document S1. Figures S1–S8 and Table S1 [file mmc1.pdf]

OMTN, Volume *28*

Supplemental information

AntimiR targeting of microRNA-134 reduces seizures in a mouse model of Angelman syndrome

Aoife Campbell, Gareth Morris, Albert Sanfeliu, Joana Augusto, Elena Langa, Jaideep C. Kesavan, Ngoc T. Nguyen, Ronan M. Conroy, Jesper Worm, Lukasz Kielpinski, Mads Aaboe Jensen, Meghan T. Miller, Thomas Kremer, Cristina R. Reschke, and David C. Henshall

Table S1: Upregulated DEGs of interest between Ant-134 and Scr-treated Ube3a mice. DEGs of interest include predicted miR-134 targets, genes previously identified as differentially expressed after Ant-134 treatment [*Reschke et al, Mol Ther 2021*][28] and genes associated with epilepsy.

Gene Symbol	log2 Fold Change	p-value	Previously identified Ant-134 target	miR-134 target	Epilepsy associated
Scn3b	0.14	0.0004	NO	NO	YES
Ar	0.23	0.0011	NO	NO	YES
Chrm1	0.11	0.0021	NO	NO	YES
Drd5	0.30	0.0036	NO	YES	NO
Ldb3	0.61	0.0042	NO	YES	NO
Gstm1	0.19	0.0043	NO	NO	YES
Arpc2	0.13	0.0053	NO	NO	YES
Gtf2h1	0.16	0.0056	NO	YES	NO
Zmynd8	0.13	0.0082	NO	NO	YES
Chn1	0.12	0.0087	NO	NO	YES
Fer	0.23	0.0109	NO	YES	NO
Calm2	0.10	0.0124	NO	NO	YES
Lman2l	0.16	0.0139	NO	NO	YES
Ccdc6	0.13	0.0164	NO	NO	YES
Grem1	0.37	0.0178	NO	YES	YES
Crb1	0.79	0.0188	NO	NO	YES
Dapk1	0.12	0.0193	NO	NO	YES
Pcp4	0.29	0.0201	NO	NO	YES
Kctd21	0.20	0.0204	NO	YES	NO
Cox10	0.14	0.0212	NO	NO	YES
Tnfsf14	0.96	0.0236	NO	NO	YES
Snap25	0.12	0.0260	NO	NO	YES
Eno2	0.10	0.0269	NO	NO	YES
Suclg1	0.11	0.0280	NO	NO	YES
Tbl1xr1	0.12	0.0290	NO	NO	YES
Ppia	0.21	0.0296	NO	NO	YES
Dlg3	0.09	0.0296	NO	NO	YES
Katnal1	0.12	0.0299	NO	YES	NO
Gatad2b	0.09	0.0304	NO	NO	YES
Hspd1	0.10	0.0305	NO	NO	YES
Nptn	0.07	0.0311	NO	NO	YES
Acadsb	0.10	0.0339	NO	NO	YES
Bhlhe22	0.17	0.0355	NO	NO	YES
Ppp3ca	0.10	0.0364	NO	NO	YES
Ndufaf1	0.18	0.0371	NO	NO	YES

Smarca2	0.08	0.0377	NO	NO	YES
Ndufa11	0.14	0.0378	NO	NO	YES
Ptgs2	0.23	0.0382	NO	NO	YES
Gabrg2	0.09	0.0390	NO	NO	YES
Npy1r	0.15	0.0402	NO	YES	NO
Snca	0.10	0.0409	NO	NO	YES
Glo1	0.09	0.0433	NO	NO	YES
Hsp90ab1	0.07	0.0441	NO	NO	YES
Ywhae	0.06	0.0445	NO	NO	YES
Gpt2	0.13	0.0470	NO	NO	YES
Eef1a2	0.08	0.0473	NO	NO	YES
Lat	0.59	0.0474	NO	NO	YES
Ier3	0.34	0.0476	YES	NO	NO
Mfsd8	0.17	0.0482	NO	NO	YES
Sgce	0.15	0.0490	NO	NO	YES
Tpo	0.55	0.0499	NO	NO	YES
Slc1a1	0.08	0.0499	NO	NO	YES

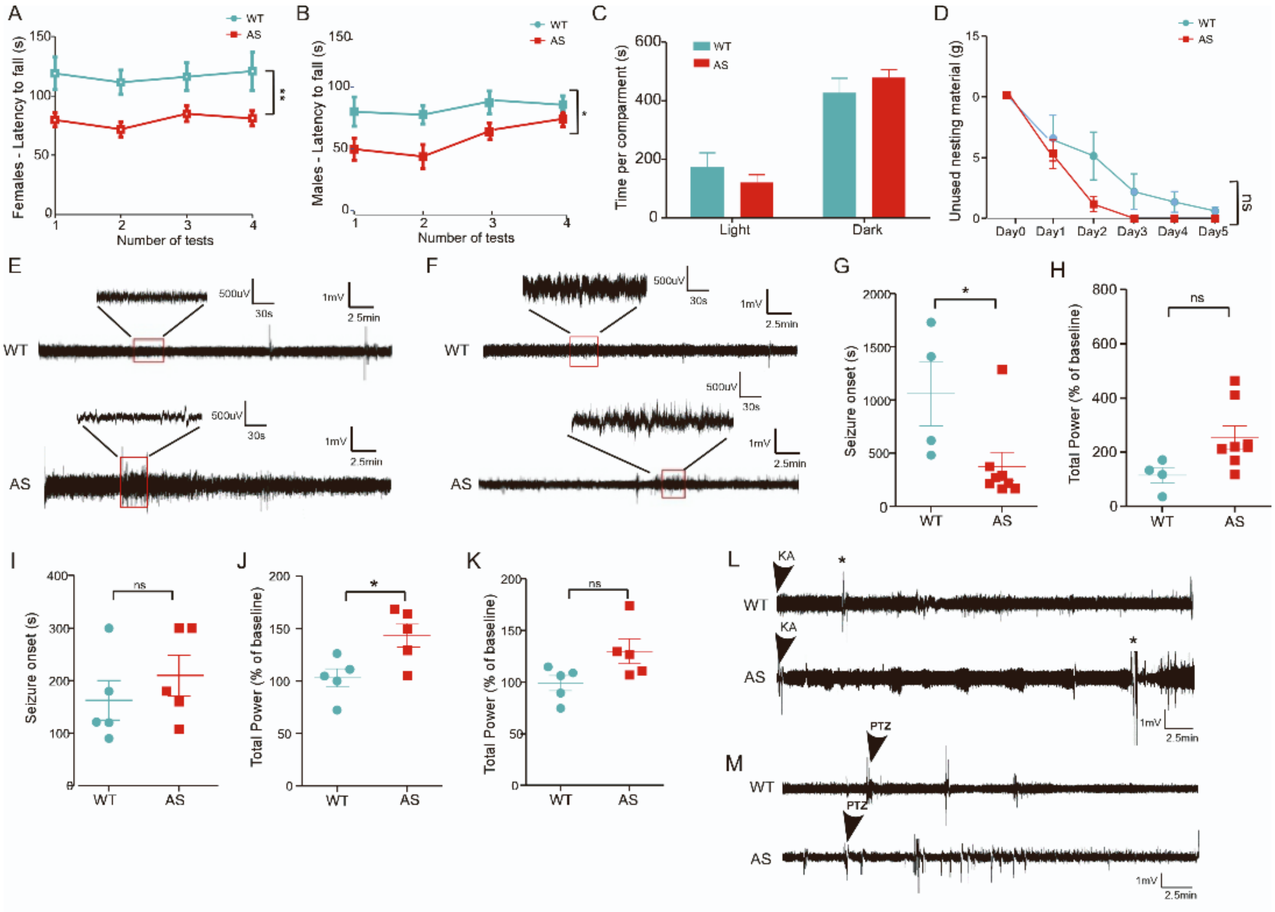

Figure S1: Additional features of F1 generation AS mice. Analysis of behavioural and seizure sensitivity phenotype in P42 F1 *Ube3a*$^{m-/p+}$ mice. **(A-B)** Graphs show the time spent on the rotarod during four test phases in **(A)** female mice, WT n=7, AS n=10, p=0.004 and **(B)** in male mice, WT n=7, AS n=10, p=0.015. **(C)** Graphs shows the length of time (s) spent in dark and light compartment during the 10 minute trial, WT n=8, AS n=14, p=0.16. **(D)** Graph shows the amount of unused nesting material from day 0 to day 5, WT n=3, AS n=4, p=0.16. **(E-F)** Representative EEG traces of 1 hour recording from **(E)** P21 and **(F)** adult WT and AS mice, Scale bar 1 mV, 2.5 min for full recording, 500 uV, 30 s for zoom. **(G)** Time (s) to the first electrographic seizure following injection of a low dose of KA (20 mg/kg, i.p.), WT *n*=4, AS *n*=8, p=0.0162. **(H)** Total power increase (%) from its own baseline when recording ictal activity following injection of 20 mg/kg KA, WT *n*=4, AS *n*=8, p=0.053. **(I)** Time (s) to the first electrographic seizure following low dose PTZ (40 mg/kg, i.p.), WT n=5, AS n=5, p=0.457. **(J)** Total power (% of increase from each mouse own baseline) from the first 10 minutes after injection 40 mg/kg PTZ, WT n=5, AS n=5, p=0.024**.** **(K)** Total power (% baseline) from entire 30 minute recording after injection 40 mg/kg PTZ, WT n=5, AS n=5, p=0.059**.** **(L-M)** Representative EEG traces of WT and AS mice following **(L)** 20 mg/kg KA or **(M)** 40 mg/kg PTZ administration (black arrows), Scale bar 1 mV, 2.5 min. The Shapiro-Wilk test was used to test for normality, and *t*-test or a Mann-Whitney test was performed for statistical analysis. Data are expressed as mean ± SEM.

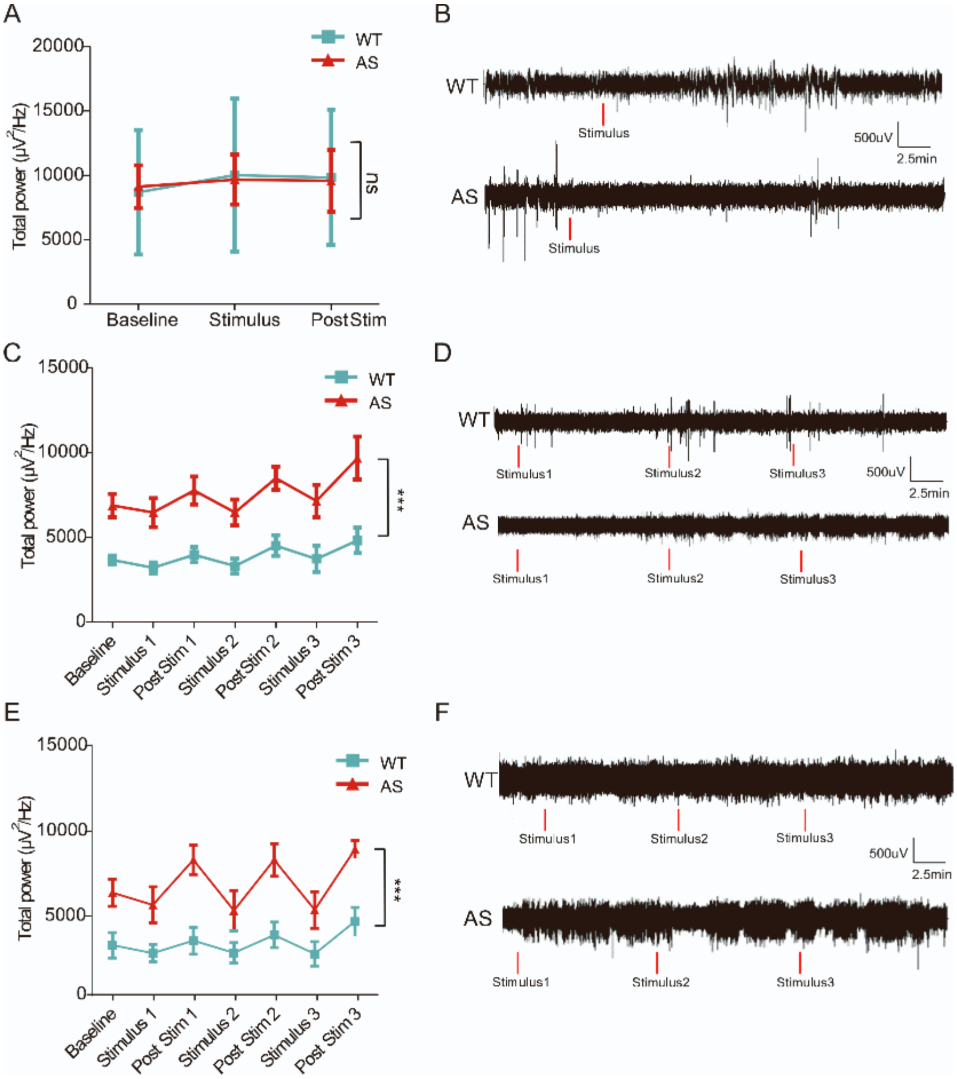

Figure S2: Inability to elicit audiogenic seizures in F1 generation AS mice. F1 WT and AS mice were used in attempt to generate an audiogenic seizure model at three different ages. **(A)** No changes in the EEG total power (μV^2Hz) at baseline, during stimulus and post-stimulus were observed in P21 mice, WT n=3, AS n=3, p=0.99. **(B)** Representative EEG traces of right hemisphere recordings from WT and AS P21 mice. **(C-F)** Although significant changes in the total EEG power (μV^2Hz) were observed at baseline, during and after stimuli in **(C)** P28 (WT *n*=4, AS *n*=8, ***p<0.001) and **(E)** in P42 mice (WT n=3, AS n=3, ***p<0.001), no audiogenic-induced clinical (behavioural) seizures were observed. Representative EEG traces of the right brain hemisphere recordings in WT and AS **(D)** P28 and **(F)** P42 mice depict pre- and post- audiogenic stimulus periods. Data are represented as mean ± SEM. A Shapiro-Wilk test was used to check normality of data and two-way ANOVA was used for statistical analysis.

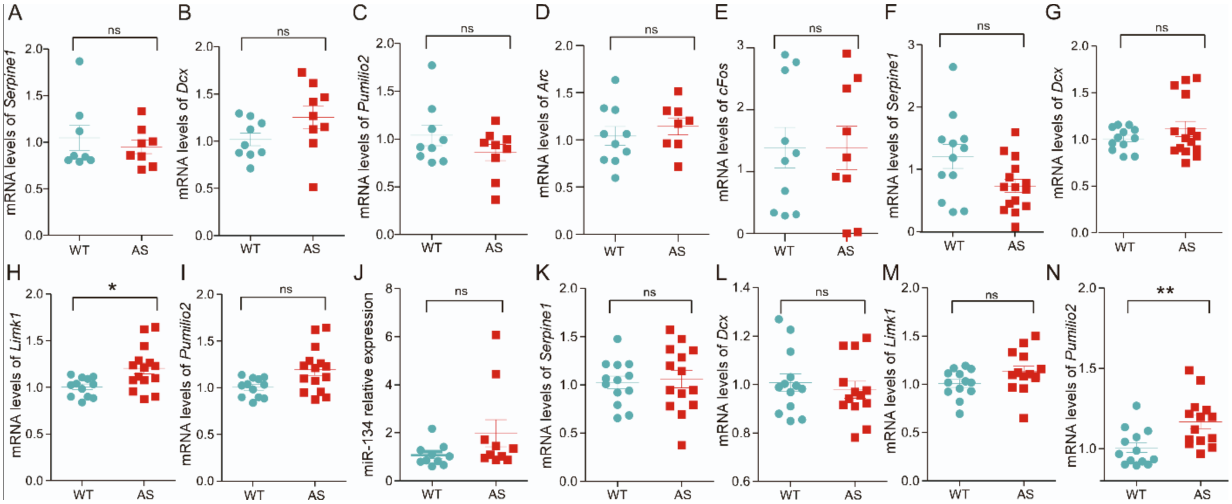

Figure S3: **Additional gene expression analyses in AS mice.** Transcript analysis of levels of miR-134 and its targets from the **(A-E)** cerebellum, **(F-I)** hippocampus and **(J-N)** cortex of naïve P21 F1 generation mice. **(A)** mRNA levels of *Serpine1* in the cerebellum of P21 mice, WT n=8, AS n=8, p=0.54. **(B)** mRNA levels of *Dcx* in the cerebellum of P21 mice, WT n=9, AS n=9, p=0.11. **(C)** mRNA levels of *Pumilio2* in the cerebellum of P21 mice, WT n=9, AS n=9, p=0.21. **(D)** mRNA levels of *Arc* in the cerebellum of P21 mice, WT n=10, AS n=9, p=0.470 **(E)** mRNA levels of *cFos* in the cerebellum of P21 mice, WT n=10, AS, n=9, p=0.99 **(F)** mRNA levels of *Serpine1* in the hippocampus of P21 mice, WT n=12, AS n=15, p=0.037. **(G)** mRNA levels of *Dcx* in the hippocampus of P21 mice, WT n=12, AS n=15, p=0.30. **(H)** mRNA levels of *Limk1* in the hippocampus of P21 mice, WT n=12, AS n=15, *p=0.01. **(I)** mRNA levels of *Pumilio2* in the hippocampus of P21 mice, WT n=12, AS n=15, p=0.02. **(J)** Relative expression of miR-134 in the cortex of P21 mice, WT n=10, AS n=10, p=0.14 **(K)** mRNA levels of *Serpine1* in the cortex of P21 mice, WT n=13, AS n=14, p=0.80. **(L)** mRNA levels of Dcx in the cortex of P21 mice, WT n=13, AS n=13, p=0.60. **(M)** mRNA levels of *Limk1* in the cortex of P21 mice, WT n=13, AS n=14, p=0.09. **(N)** mRNA levels of *Pumilio2* in the cortex of P21 mice, WT n=13, AS n=14, **p=0.005. U19 was used as a normaliser for miR-134 levels. β-actin was used as a normaliser for protein coding transcript analysis. A t-test was used for statistical analysis and a Shapiro-Wilk test was used to test for normality. Multiple corrections were performed on molecular data and α was adjusted to 0.0125. Data are expressed as mean ± SEM.

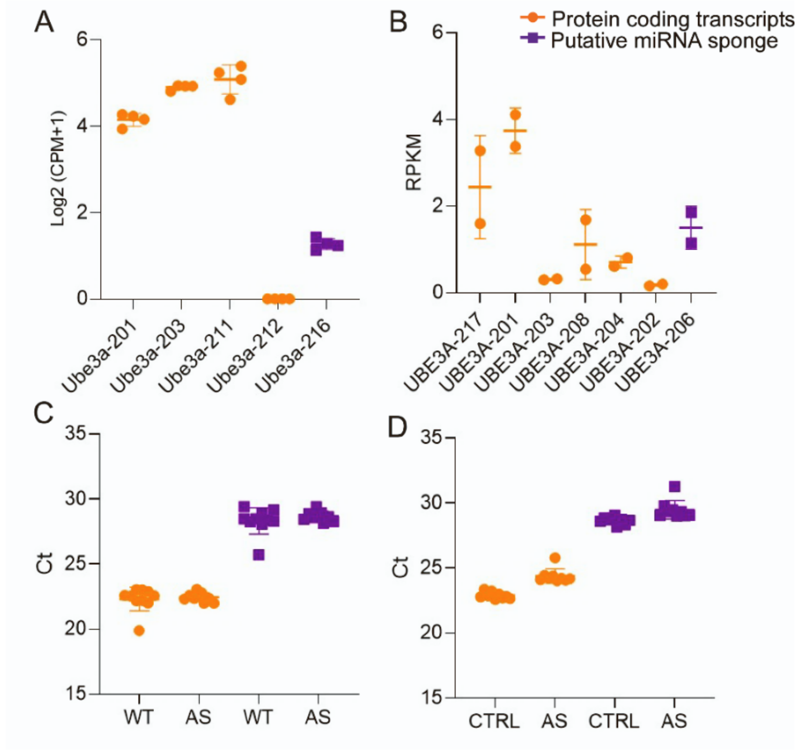

Figure S4: **Expression levels of the Ube3a putative miRNA sponge splicing variant relative to its protein coding variants. (A-B)** Graphs depict representative RNAseq dataset retrieved from the Gene Expression Omnibus (GEO) database for the levels of specific Ube3a/UBE3A splicing variants in the **(A)** hippocampus of WT mice (n=4), and in **(B)** human cortex samples (n=2)**. (C-D)** Graphs show the expression of the selected Ube3a/UBE3A splicing variants measured by RT-qPCR in the **(C)** hippocampi of P24 WT (n=10) and Ube3am$^{-/p+}$ mice (n=9), along with **(D)** control (n=9) and AS (n=9) human iPSCs. Ct= Cycle threshold, CPM=Counts Per Million, RPKM=Reads Per Kilobase per Million.

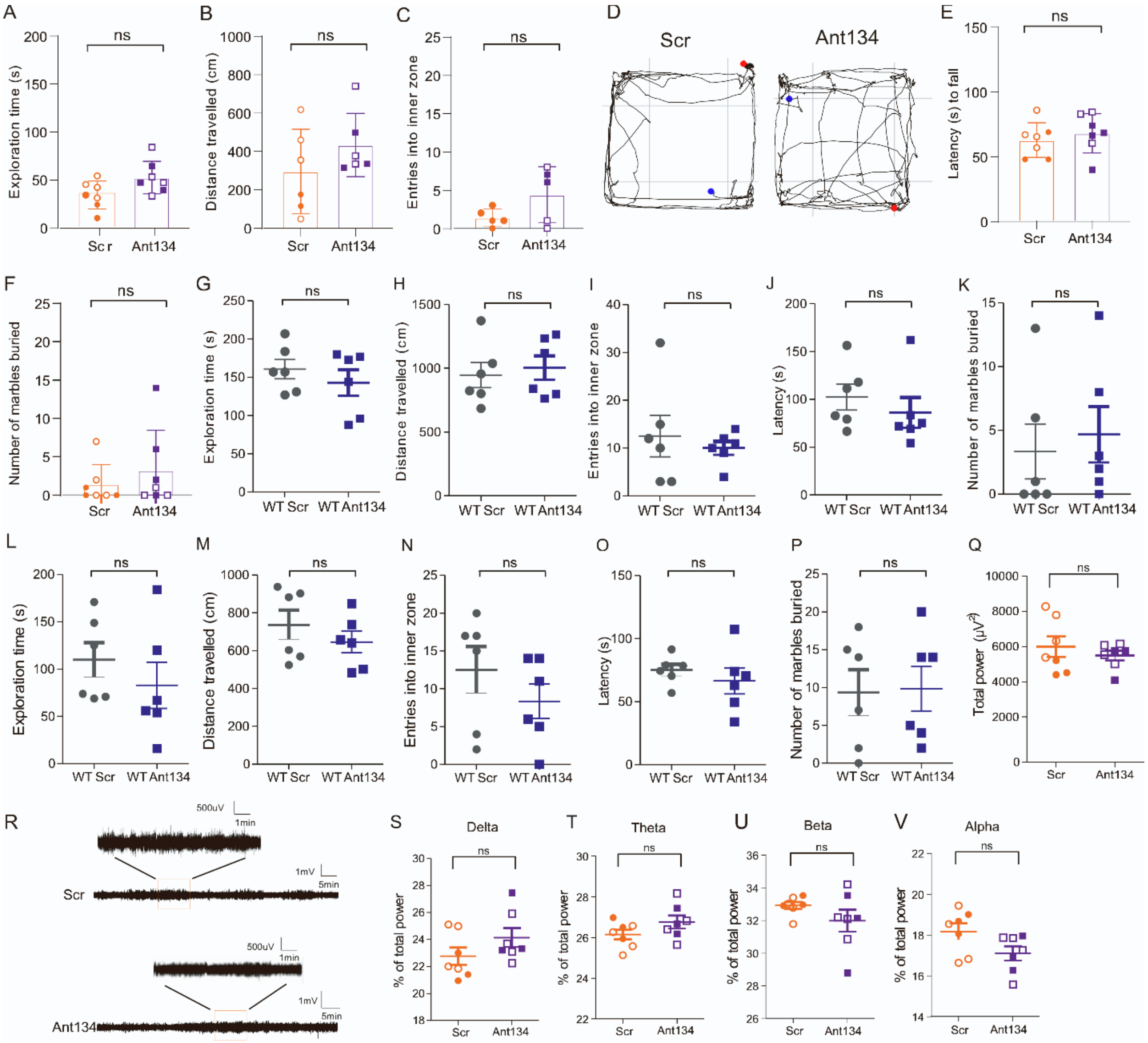

Figure S5: **Re-testing Ant-134-treated F1 AS mice and effects of Ant-134 in wildtype mice.** **(A-F)** Ant-134/Scr-treated F1 AS mice were re-tested 3 weeks later at P42 and put through the behavioural tests. **(A)** Exploration time (s) during open field in P42 mice, Scr n=7, Ant-134 n=7, p=0.053. **(B)** Distance travelled (cm) in open field arena, Scr n=6, Ant-134, n=6, p=0.245. **(C)** Entries into inner zone of open field arena, Scr n=5, Ant-134 n=5, p=0.117. **(D)** Representative track plot of Scr/Ant-134 mouse at P42 in open field. **(E)** Latency to fall (s) on rotarod, Scr n=7, Ant-134 n=7, p=0.50. **(F)** Number of marbles buried, Scr n=7, Ant-134 n=7, p=0.60. **(G-K)** In a separate experiment, P21 WT mice were given Scr/Ant-134 and 24 h later put through behavioural tests. **(G)** Exploration time (s) during open field, Scr n=6, Ant-134 n=6, p=0.43**. (H)** Distance travelled (cm) in open field arena, Scr n=6, Ant-134 n=6, p=0.68. **(I)** Entries into inner zone of open field arena, Scr n=6, Ant-134 n=6, p=0.60. **(J)** Latency to fall (s) on rotarod, Scr n=6, Ant-134 n=6, p=0.45. **(K)** Number of marbles buried, Scr n=6, Ant-134 n=6, p=0.67. **(L-V)** Re-testing of WT mice at P42 after treatment with Scr/Ant-134 at P21. **(L)** Exploration time (s) during

open field in P42 WT mice, Scr n=6, Ant-134 n=6, p=0.40. **(M)** Distance travelled (cm) in open field arena in P42 WT mice, Scr n=6, Ant-134 n=6. **(N)** Entries into inner zone of open field arena in P42 WT mice, Scr n=6, Ant-134 n=6, p=0.30. **(O)** Latency to fall (s) on rotarod in P42 WT mice, Scr n=6, Ant-134 n=6, p=0.45. **(P)** Number of marble buried in P42 WT mice, Scr n=6, Ant-134 n=6, p=0.91. The effect of Ant-134 on baseline EEG and behavioural phenotypes in F1 mice was analysed. P42 mice were pre-treated with Scr/Ant-134 and resting EEG was recorded 24 h later. **(Q)** Total power (μV^2) of baseline EEG in Scr and Ant-134 mice, n=7/group, p=0.45. **(R)** Representative EEG traces for Scr and Ant-134 mice of 1 h recordings, scale bar 1 mV, 5 min. Zoom scale bar shows 10 minute recording, 500 uV, 1 min. **(S)** % of total power of delta, n=7/group, p=0.17. **(T)** % of total power of theta, n=7/group, p=0.14. **(U)** % of total power of beta, n=7/group, p=0.21. **(V)** % of total power of alpha, n=7/group, p=0.06.

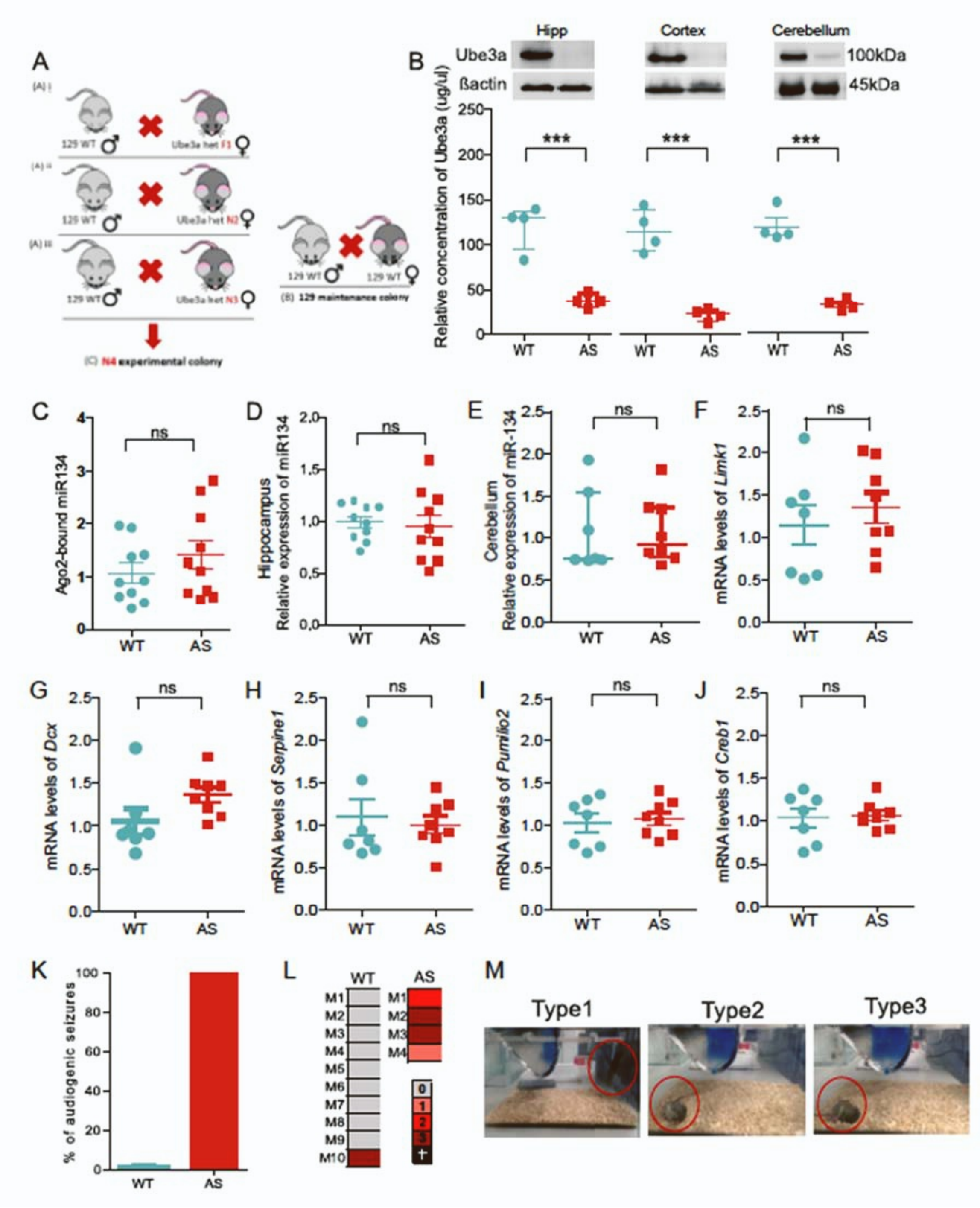

Figure S6: Generation of AS mice susceptible to audiogenic seizures. Generation of N4 colony AS mice susceptible to audiogenic seizures. **(A)** Breeding scheme of N4 colony by backcrossing from generation F1 *(Ai)* to generate N3 female mice *(Aiii)*. N3 female mice were crossed with the 129 maintenance colony *(Aii)* to yield the N4 experimental colony *(Aiv)*. **(B&C)** Western blot showing protein levels of Ube3a in the hippocampus, cortex and cerebellum of P21 N4 mice. Semi-quantification of Ube3a levels in the hippocampus (WT n=4, AS n=4, ***p=0.0008),

cortex (WT n=4, AS n=4 ***p=0.0003) and cerebellum (n=4 WT, AS n=4, ***p=0.0286). **(C)** Relative expression of Ago-2 bound miR-134 in the hippocampus at P21, WT n=10, AS n=10, p=0.312. **(D)** Relative expression of miR-134 in the hippocampus of N4 P21 mice, WT n=10, AS n=10 p= 0.723. **(E)** Relative expression of miR-134 in the cerebellum of N4 P21 mice, WT n=7, AS n=8, p=0.693. **(F)** mRNA levels of *limk1* in the cerebellum of N4 P21 mice,WT n=7, AS n=8, p=0.5139. **(G)** mRNA levels of *Dcx* in the cerebellum of N4 P21 mice, WT n=7, AS n=8, p=0.09. **(H)** mRNA levels of *Serpine1* in the cerebellum of N4 P21 mice, WT n=7, AS n=8, p=0.693. **(I)** mRNA levels of *Pumilio2* in the cerebellum of N4 P21 mice, WT n=7, AS n=8, p=0.709. **(J)** mRNA levels of *Creb1* in the cerebellum of N4 P21 mice, WT n=7, AS n=8, p=0.796. **(K)** N4 P21 AS mice are susceptible to audiogenic-induced seizures (WT n=10, AS n=4). **(L)** Individual seizure characterization using a scoring scale for audiogenic seizures. Type 0: No seizure activity, Type 1: Wild running when stimulus is initiated, Type 2: Generalized tonic-clonic convulsions with loss of posture, Type 3: Tonic hyperextension of the hindlimbs and tail and Type 4: Death. **(M)** Screenshots from videos recording elicited audiogenic seizures and covering examples of types 1-3. Data are expressed as SEM or median and interquartile range. A *t*-test or a Mann-Whitney test was used for analysis between WT and AS mice.

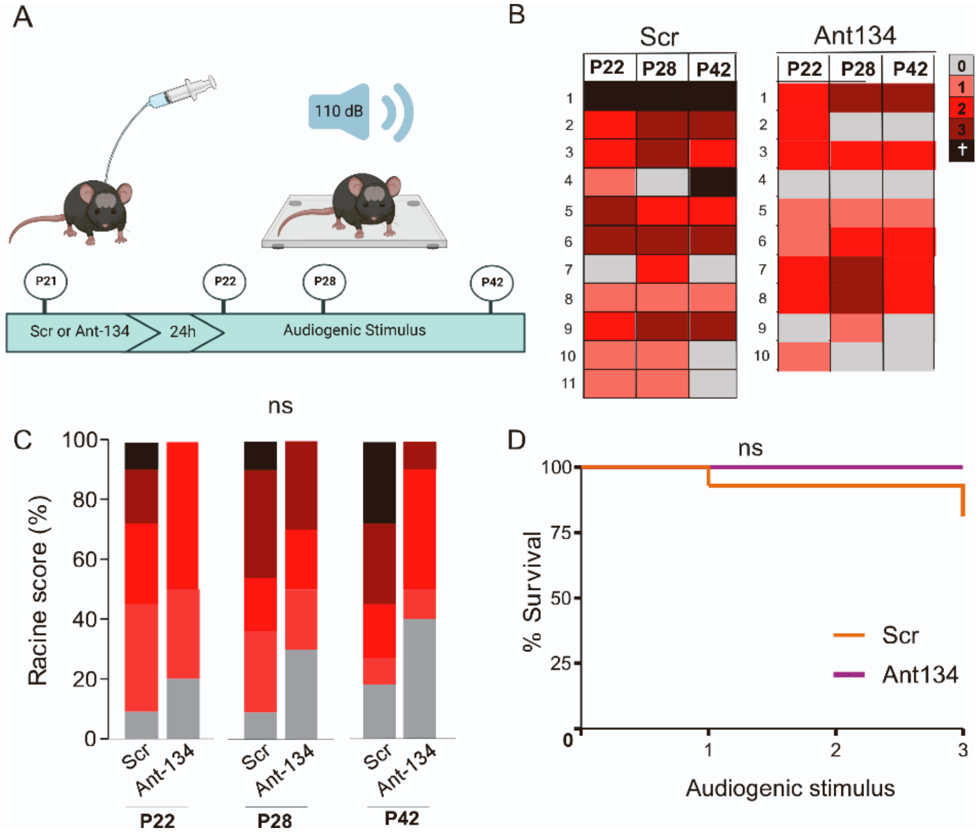

Figure S7: Assessment of longer-term effects of Ant-134 on audiogenic-induced seizures. **(A)** N4 P21 AS mice were pre-treated with 0.5 nmol/2µl of Ant-134 or Scr (ICV) and subjected to three individual audiogenic stimuli at P22, then at P28 and finally at P42. Image drawn using Biorender. **(B)** Tabular representation of seizure severity per mouse per time point following Scr/Ant-134 (0.5 nmol/2µl; i.c.v.; Scr n=11, Ant-134 n=10). **(C)** Scale representing seizure severity (% per type 0-4) over the course of three time points (P22, P28 and P42; Scr n=11, Ant-134 n=10, p=0.22). **(D)** Percentage of survival of mice pre-treated with Scr/Ant-134 (0.5 nmol) over the course of three seizures, Scr n=11, Ant-134 n=10. Data were analysed with Stata Release 16.1. Mortality was compared between treatments using incidence rate ratios and a Poisson model, with number of trials as the exposure variable. Seizure severity was modelled using ordinal logistic regression with robust variance estimation used to adjust for clustering of data with mice. An interaction term was used to test for a change in effectiveness of treatment as a function of trial number. For the survival curve analysis, a Log-rank (Mantel-Cox) test was used.

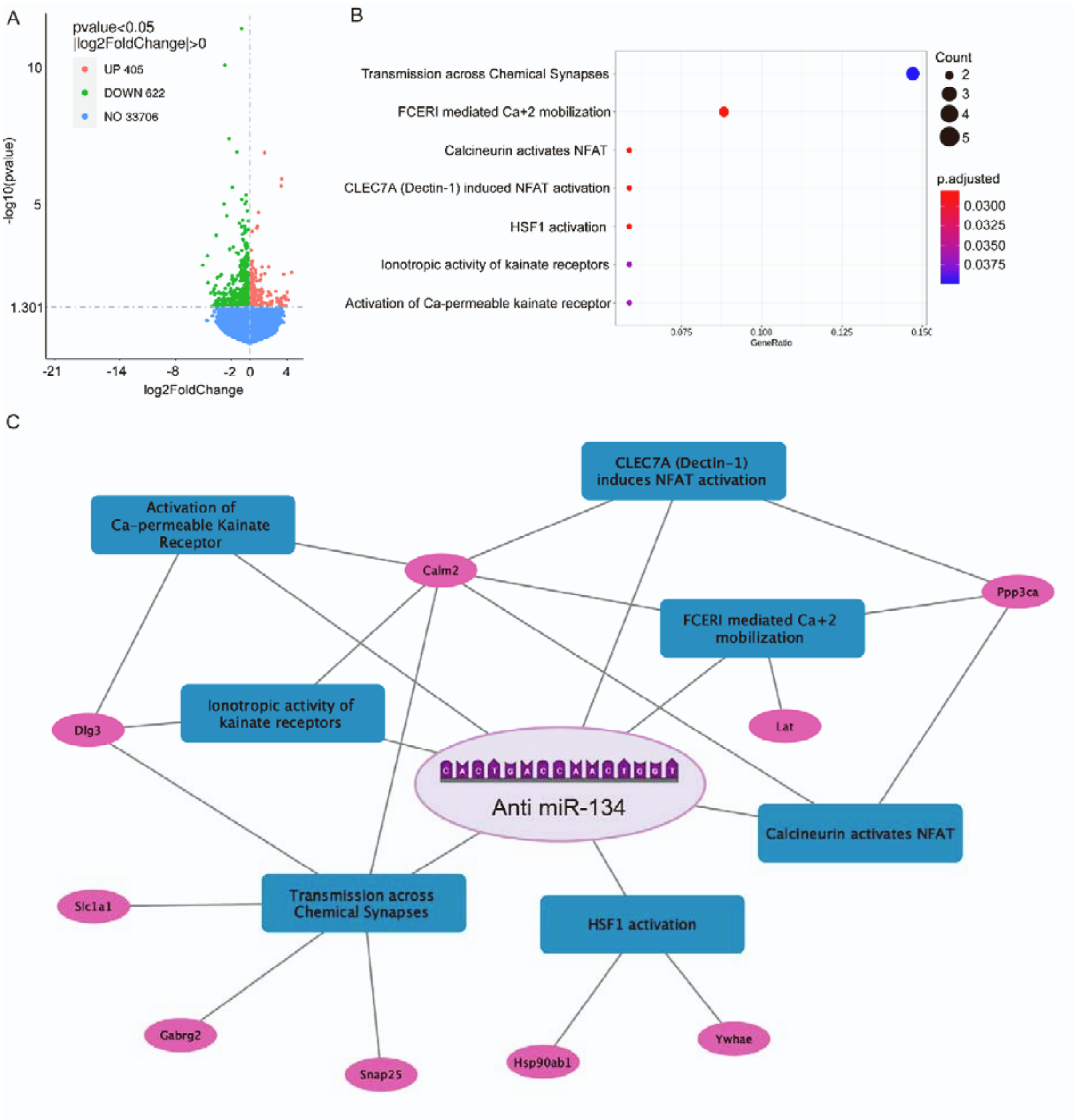

Figure S8: Gene expression profile after Ant-134 treatment in AS mice. (**A**) Volcano plot of the Differentially Expressed Genes (DEGs) between Ant-134 (n=5) and Scr-treated (n=4) AS mice identified using RNAseq. Genes with a p-value <0.05 and a |Log2 Fold Change| > 0 were considered differentially expressed. 405 genes were upregulated and 622 genes were downregulated in the Ant-134 mice. **(B)** Reactome Pathways enriched among DEGs of interest. Reactome pathway enrichment was performed on the list of DEGs of interest. Pathways with an adjusted p-value <0.05 were considered significantly enriched. The Gene Ratio is the number of DEGs of interest annotated to a given pathway over the total genes annotated to the same pathway. The Background Ratio is the number of total genes annotated to a given pathway over the total number of genes represented in the Reactome database. **(C)** Reactome Pathways enriched among DEGs of interest. Network of the significantly enriched Reactome Pathways and their associated DEGs of interest.

Supplementary movie titles and legends

SI Movie M1

Representative video of a P22 AS mouse in the open field that had been treated 24 h earlier with scrambled.

SI Movie M2

Representative video of a P22 AS mouse in the open field that had been treated 24 h earlier with Ant-134.

SI Movie M3

Representative video of a P22 AS mouse response to audiogenic seizure stimulus that had been treated 24 h earlier with scrambled.

SI Movie M4

Representative video of a P22 AS mouse response to audiogenic seizure stimulus that had been treated 24 h earlier with Ant-134.

SI Movie M5

Movie showing spontaneous network activity in Fluo-4 loaded neurons differentiated from Angelman iPSC line. The fluorescence variation is shown in pseudo-colours. Scale bar: 30 µm.

Reference

28. Reschke, C.R., Silva, L.F.A., Vangoor, V.R., Rosso, M., David, B., Cavanagh, B.L., Connolly, N.M.C., Brennan, G.P., Sanz-Rodriguez, A., Mooney, C. et al. (2021). Systemic delivery of antagomirs during blood-brain barrier disruption is disease-modifying in experimental epilepsy. Mol. Thera. *29*, 2041-2052.
