# Supplementary material for: AntimiR targeting of microRNA-134 reduces seizures in a mouse model of Angelman syndrome
Source: Mol Ther Nucleic Acids. 2022 Apr 20;28:514–29. doi: 10.1016/j.omtn.2022.04.009 (PMC9092865; doi:10.1016/j.omtn.2022.04.009)
Supplement: Document S2. Article plus supplemental information [file mmc7.pdf]

Molecular Therapy
Nucleic Acids
Original Article

AntimiR targeting of microRNA-134 reduces seizures in a mouse model of Angelman syndrome

Aoife Campbell,[1,2] Gareth Morris,[1,2] Albert Sanfeliu,[1,2] Joana Augusto,[1,2] Elena Langa,[1,2] Jaideep C. Kesavan,[1,2] Ngoc T. Nguyen,[1,2] Ronan M. Conroy,[3] Jesper Worm,[4] Lukasz Kielpinski,[4] Mads Aaboe Jensen,[4] Meghan T. Miller,[5] Thomas Kremer,[5] Cristina R. Reschke,[1,2,6,7] and David C. Henshall[1,2,7]

[1]Department of Physiology and Medical Physics, RCSI University of Medicine and Health Sciences, Dublin D02 YN77, Ireland; [2]FutureNeuro, The SFI Research Centre for Chronic and Rare Neurological Diseases, RCSI University of Medicine and Health Sciences, Dublin D02 YN77, Ireland; [3]Department of Public Health and Epidemiology, RCSI University of Medicine and Health Sciences, Dublin D02 YN77, Ireland; [4]Therapeutic Modalities, Roche Innovation Center Copenhagen A/S, F. Hoffmann-La Roche Ltd, DK-2970 Hørsholm, Denmark; [5]Neuroscience and Rare Diseases Discovery and Translational Area, Roche Innovation Center Basel, F. Hoffmann-La Roche Ltd, CH-4070 Basel, Switzerland; [6]School of Pharmacy and Biomedical Sciences, RCSI University of Medicine and Health Sciences, Dublin D02 YN77, Ireland

Angelman syndrome (AS) is a severe neurodevelopmental disorder featuring ataxia, cognitive impairment, and drug-resistant epilepsy. AS is caused by mutations or deletion of the maternal copy of the paternally imprinted *UBE3A* gene, with current precision therapy approaches focusing on re-expression of *UBE3A*. Certain phenotypes, however, are difficult to rescue beyond early development. Notably, a cluster of microRNA binding sites was reported in the untranslated *Ube3a1* transcript, including for miR-134, suggesting that AS may be associated with microRNA dysregulation. Here, we report levels of miR-134 and key targets are normal in the hippocampus of mice carrying a maternal deletion of *Ube3a* (*Ube3a*$^{m-/p+}$). Nevertheless, intracerebroventricular injection of an antimiR oligonucleotide inhibitor of miR-134 (Ant-134) reduced audiogenic seizure severity over multiple trials in 21- and 42-day-old AS mice. Interestingly, Ant-134 also improved distance traveled and center crossings of AS mice in the open-field test. Finally, we show that silencing miR-134 can upregulate targets of miR-134 in neurons differentiated from Angelman patient-derived induced pluripotent stem cells. These findings indicate that silencing miR-134 and possibly other microRNAs could be useful to treat clinically relevant phenotypes with a later developmental window in AS.

INTRODUCTION

Angelman syndrome (AS; Online Mendelian Inheritance in Man [OMIM] 105830) is a severe neurodevelopmental disorder that affects 1 in 12,000–20,000 male and female patients. The clinical symptoms include profound cognitive impairment, ataxia, and drug-resistant epilepsy.[1,2] AS is caused by deficiency of the *UBE3A* gene (15q11.2 in humans), which encodes an abundantly expressed ubiquitin ligase essential for synaptic structure and function.[1] *UBE3A* is an imprinted gene, whereby the paternally inherited copy is normally silenced in neurons. An inherited or *de novo* mutation or chromosomal deletion of the maternal copy results in AS.[1,3]

The therapeutic needs of people with AS are largely unmet.[3] Existing treatments include combinations of medicines that are intended to reduce some of the most severe symptoms of AS and include management of epilepsy. Gene and precision therapy (e.g., viral vectors, antisense oligonucleotides [ASOs]) hold the promise of specific, targeted correction of AS disease mechanisms. A leading approach is to "unsilence" the paternally imprinted copy of *UBE3A*, for example by targeting the long non-coding RNA that directs the imprinting process.[4-7] However, mitigation of certain phenotypes by *UBE3A* gene reactivation becomes less effective once critical developmental periods have passed.[5,8,9] This has led to additional therapeutic strategies that use alternative mechanisms while offering a protracted or open therapeutic window.[10]

It was recently reported that one of the transcripts expressed from the *Ube3a* locus in rodents, *Ube3a1*, is activity regulated, is enriched in dendrites, and has a 3′ untranslated region containing a series of complementary sites for multiple microRNAs (miRNAs) from the evolutionarily conserved miR-379∼410 cluster.[11] This raises the possibility that *Ube3a1* has protein coding-independent functions as a competing endogenous RNA or miRNA sponge.[11] miRNAs are small non-coding RNAs that negatively regulate protein levels by sequence-specific targeting of mRNAs.[12] Target search and repression occur upon loading of the miRNA into a binding pocket of an Argonaute (Ago) protein to form the miRNA-induced silencing complex (RISC).[13] Loss of transcription from the *Ube3a* locus would potentially result in imbalances or increased availability of miRNAs from the miR-379∼410, cluster leading to over-suppression of their targets.

Received 17 August 2021; accepted 15 April 2022;
https://doi.org/10.1016/j.omtn.2022.04.009.

[7]These authors contributed equally

Correspondence: Cristina R. Reschke, PhD, School of Pharmacy and Biomedical Sciences, RCSI University of Medicine and Health Sciences, 123 St. Stephen's Green, Dublin D02 YN77, Ireland.
E-mail: cristinarreschke@rcsi.ie
Correspondence: David C. Henshall, PhD, Department of Physiology and Medical Physics, RCSI University of Medicine & Health Sciences, 123 St. Stephen's Green, Dublin D02 YN77, Ireland.
E-mail: dhenshall@rcsi.ie

 © 2022 The Author(s).
This is an open access article under the CC BY license (http://creativecommons.org/licenses/by/4.0/).

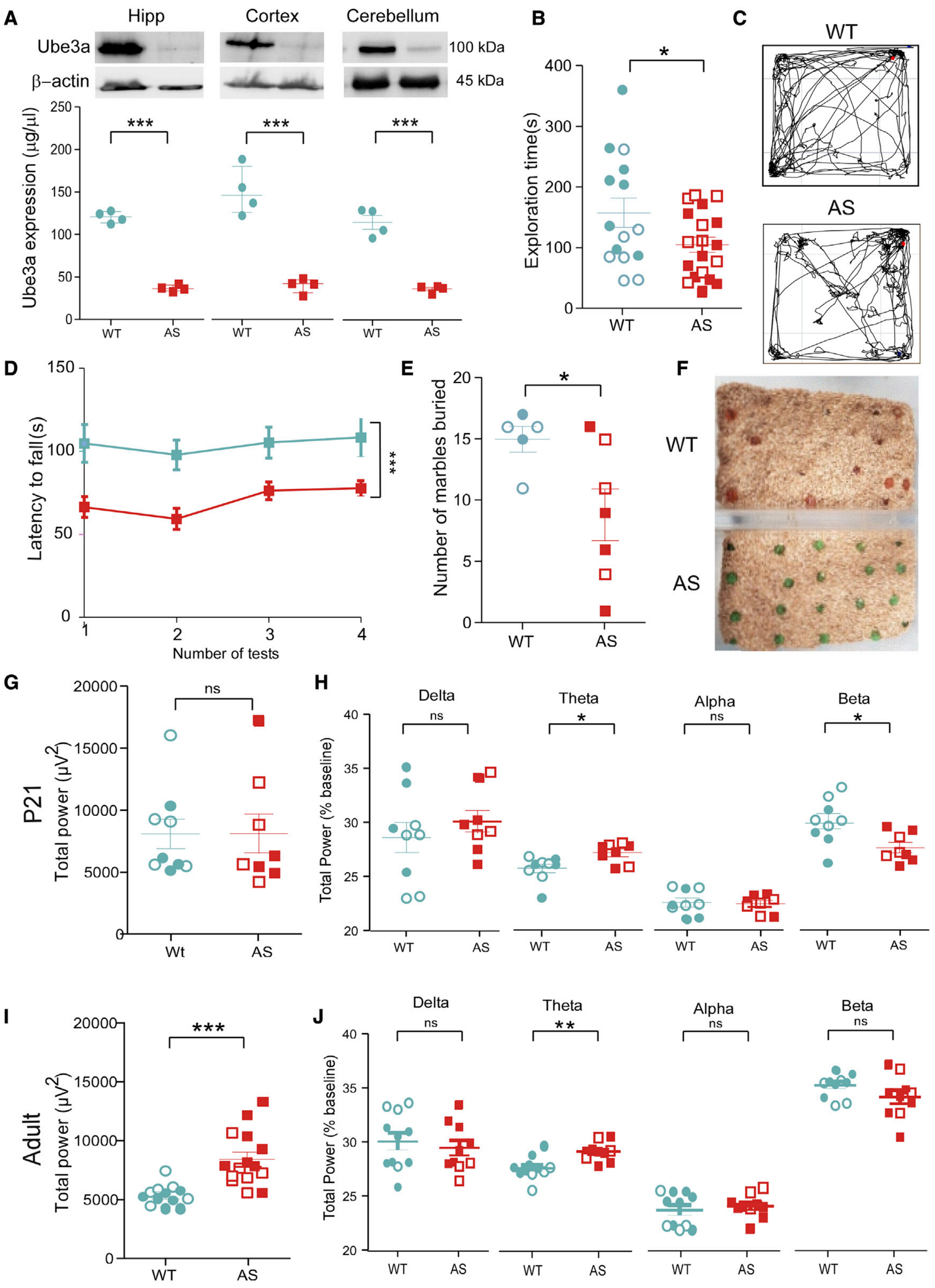

Figure 1. *Ube3a$^{m-/p+}$* mice model multiple AS phenotypes

(A) Lack of Ube3a (E6-AP) confirmed by western blotting lysates from P21 F1 *Ube3a* $^{(m-/p+)}$ (AS) mice compared with wild-type (WT) littermate controls. n = 4/genotype; ***p < 0.001. (B) Total time (s) spent exploring the open-field arena over a 10 min trial in P21 mice; WT, n = 15; AS, n = 19; *p = 0.022. (C) Representative track plot from each

(legend continued on next page)

Notably, one of the conserved sites is for miR-134, which is upregulated in experimental and human drug-resistant epilepsy[14-17] and is a biomarker of cognitive impairment.[18] Experimentally validated targets of miR-134 include the dendritic spine regulator LIM kinase1 (*Limk1*),[19] transcription factor *Creb1*,[20] and neuronal migration factor doublecortin (*Dcx*).[21] Reducing brain levels of miR-134 using modified ASOs termed antimiRs suppresses both evoked and spontaneous seizures in rodents.[14,16,22-24] AntimiRs against miR-134 were also been reported to restore learning and synaptic plasticity in mice lacking *Sirtuin1*.[20] This suggests a mechanistic link between miR-134 and the molecular pathogenesis of AS, raising the possibility of a therapeutic approach targeting this miRNA. Here, we investigated the expression of the *Ube3a1* transcript, miR-134 and its targets, and tested the effects of miR-134 inhibition *in vivo* in AS mice.

RESULTS

F1 *Ube3a*$^{m-/p+}$ mice model multiple AS phenotypes

To investigate whether targeting miR-134 could alleviate phenotypes in AS mice, we bred B6.129S7-Ube3a$^{tm1alb/J}$ mice carrying a mutation in *Ube3a* on a mixed 129/C57 background.[25] Western blot analysis of protein lysates from juvenile (postnatal day 21 [P21]) first generation (F1) *Ube3a*$^{m-/p+}$ mice confirmed the lack of Ube3a protein in the hippocampus, cortex, and cerebellum (Figure 1A). *Ube3a*$^{m-/p+}$ mice display cognitive, locomotor, electroencephalography (EEG), and seizure susceptibility phenotypes.[6,8,26] Consistent with these reports, we detected multiple AS-like features in P21 F1 *Ube3a*$^{m-/p+}$ mice of both sexes compared with wild-type littermates. This included reduced exploration in the open-field test (Figures 1B and 1C) and reduced latency to fall on the rotarod (Figures 1D, S1A, and S1B). F1 *Ube3a*$^{m-/p+}$ mice also displayed impaired natural behavior in the marble-burying assay (Figures 1E and 1F) but performed normally in the light-dark box, a measure of anxiety, and nest building compared with wild-type mice (Figures S1C and S1D).

F1 *Ube3a*$^{m-/p+}$ mice displayed modest age-related differences in resting-state EEG, including differences in the relative power of frequency bands in the theta (4–8 Hz) and beta (15–30 Hz) range in P21 mice (Figures 1G, 1H, and S1E). In older (P42) F1 *Ube3a*$^{m-/p+}$ mice, there was an increase in raw broadband EEG power (Figure 1I) and an increased relative power in the theta frequency band (Figures 1J and S1F). We did not detect the reported delta frequency abnormality.[27] P42 and older F1 *Ube3a*$^{m-/p+}$ mice also displayed sensitivity to low doses of systemically administered chemoconvulsants (Figures S1G–S1M). We did not observe spontaneous seizures and were unable to elicit audiogenic seizures in juvenile F1 *Ube3a*$^{m-/p+}$ mice (Figure S2). Taken together, these results indicate juvenile and older F1 *Ube3a*$^{m-/p+}$ mice display many of the expected phenotypes associated with AS.

miR-134 and target transcript levels in F1 *Ube3a*$^{m-/p+}$ mice

To test the hypothesis that levels of miR-134 in *Ube3a*$^{m-/p+}$ mice may be altered, we extracted RNA from brain regions relevant to the ataxia/motor, behavioral, and EEG phenotypes (hippocampus, neocortex, and cerebellum) and performed TaqMan individual miRNA assays.[23,28] Juvenile P21 F1 *Ube3a*$^{m-/p+}$ mice displayed significant elevations in levels of miR-134 in the cerebellum (Figure 2A). Transcript levels of the validated miR-134 target *Limk1* were, however, similar to those in wild-type mice (Figure 2B). A selection of other validated miR-134 targets including *Creb1*, *Dcx*, RNA-binding protein *pumilio 2*,[29] and *Serpine1*, a predicted target of miR-134 and negative regulator of the pro-epileptogenic factor tPA,[30] also did not show differences in cerebellar samples from F1 *Ube3a*$^{m-/p+}$ mice (Figures S3A–S3C). The elevated miR-134 was unlikely to be due to a difference in overall neuronal activity levels, because cerebellar levels of two activity-regulated genes, *c-Fos* and *Arc*, were similar compared with wild-type littermates (Figures S3D and S3E).

In the hippocampus, levels of miR-134 in juvenile F1 *Ube3a*$^{m-/p+}$ mice were not significantly different compared with wild-type littermates (Figure 2C), and the majority of miR-134 target transcript levels were similar between *Ube3a*$^{m-/p+}$ mice and wild-type mice (Figures S3F–S3I). Similar findings were obtained in the neocortex of juvenile F1 *Ube3a*$^{m-/p+}$ mice where miR-134 levels were not different from wild-type, and most targets of miR-134 were expressed normally (Figures S3J–S3N).

Measurement of Ago-loaded miRNAs can provide an assessment of functionally engaged miRNAs and targeting potency.[17,31] As the hippocampus did not show a difference in total miR-134 levels, we explored if F1 *Ube3a*$^{m-/p+}$ mice had altered levels of miR-134 in the RISC. As before,[14,17] Argonaute 2 (Ago2) was eluted from the hippocampus of juvenile F1 *Ube3a*$^{m-/p+}$ mice followed by RNA extraction and measurement of miR-134 levels. Levels of Ago2-bound miR-134 were not different in the hippocampus of F1 *Ube3a*$^{m-/p+}$ mice compared with wild-type (Figure 2D).

Finally, we measured the levels of specific *Ube3a*/*UBE3A* splicing variants. First, we retrieved publicly available RNA sequencing data, which revealed the expression of the protein coding transcripts and

genotype in the open field. (D) Latency to fall from the rotarod during four test phases; WT, n = 12; AS, n = 18; ***p = 0.0008. (E) Number of marbles buried over a 30 min trial; WT, n = 5; AS, n = 7; *p = 0.045. (F) Representative image of cages 30 min after marbles trial. (G) Total power of baseline EEG over 4–6 h recordings in F1 P21 mice; WT, n = 9; AS, n = 8; p = 0.95. (H) Spectral analysis of individual band frequencies over baseline recordings. WT, n = 9; AS, n = 8; delta (1–4 Hz), p = 0.5; theta (4–8 Hz), *p = 0.015; alpha (8–12 Hz), p = 0.81; beta (12–30 Hz); *p = 0.011. (I) Total power of baseline EEG recording over 4–6 h in F1 P42 mice; WT, n = 13; AS, n = 15; ***p < 0.0001. (J) Spectral analysis of individual band frequencies over baseline recordings, n = 11/genotype. Delta (1–4 Hz), p = 0.6; theta (4–8 Hz), **p = 0.003; alpha (8–12 Hz), p = 0.7; beta (12–30 Hz), p = 0.14. Data are expressed as SEM. A Shapiro-Wilk test was used to test for normality. Open symbols represent female mice and closed symbols represent male mice. ns, non-significant.

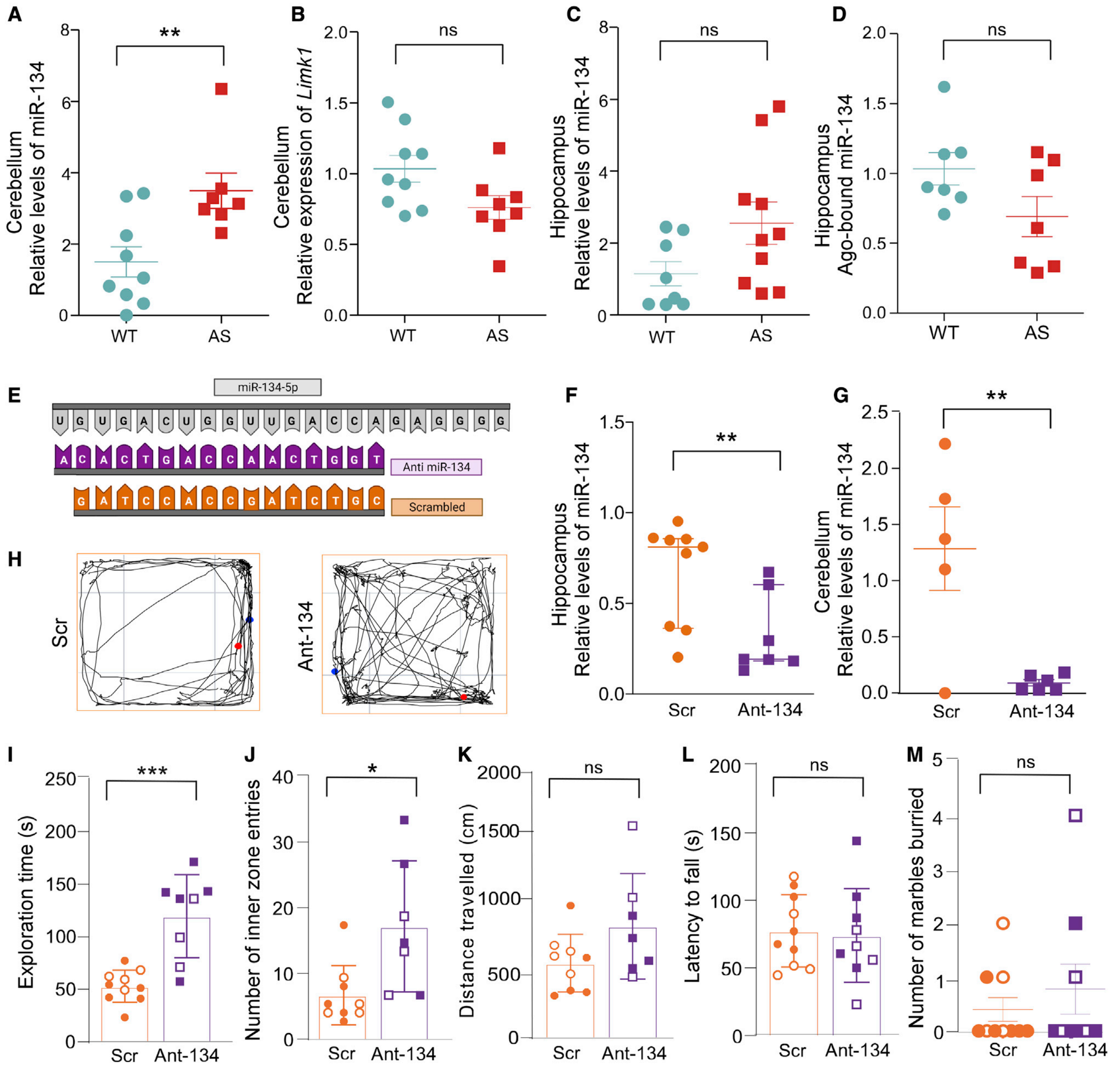

Figure 2. miR-134 levels and effects of Ant-134 in the open field in F1 *Ube3a*$^{m-/p+}$ mice

(A) Expression of miR-134 in the cerebellum of P21 mice; WT, n = 9; AS, n = 7; **p = 0.008. (B) *Limk1* in the cerebellum of P21 mice; WT, n = 9; AS, n = 8; p = 0.048. (C) miR-134 in the hippocampus of P21 mice; WT, n = 8; AS, n = 10; p = 0.069. (D) Ago-bound miR-134 in the hippocampus of P21 mice; n = 10/genotype, p = 0.209. (E) Mature miR-134 sequence and alignment of Ant-134 and scramble (Scr). (F) miR-134 levels in the hippocampus 24 h after ICV injection of Ant-134 or Scr (0.1 nmol); Scr, n = 4; Ant-134, n = 5; *p = 0.0174. (G) miR-134 levels in the cerebellum of F1 mice 24 h after Ant-134/Scr treatment (0.1 nmol); Scr, n = 5; AS, n = 4; **p = 0.006. (H) Representative track plots of F1 *Ube3a*$^{m-/p+}$ mice 24 h after treatment at P21 with either Scr or Ant-134 (0.1 nmol). (I) Exploration time (s) during open field; Scr, n = 10; Ant-134, n = 8; ***p = 0.0002. (J) Number of entries into inner zone of open field; Scr, n = 9; Ant-134, n = 7; *p = 0.01. (K) Distance traveled (cm) in open-field arena in P21 mice; Scr, n = 9; Ant-134, n = 7; p = 0.09. (L) Latency to fall (s) on accelerating rotarod; Scr, n = 10; Ant-134, n = 9; p = 0.81. (M) Number of marbles buried during 30 min marble-burying test; Scr, n = 10; Ant-134, n = 9; p = 0.75. A t test was used for analysis between Scr- and Ant-134-treated mice. Data are expressed as SEM except in F (median with interquartile range). Open symbols represent female mice and closed symbols represent male mice. Multiple corrections were performed on molecular data and α was adjusted to 0.01. ns, non-significant.

the putative miRNA sponge. Analysis found detectable levels of *Ube3a1*, albeit much lower than *Ube3a2/3* (Figures S4A and S4B). In order to validate this finding, we designed primers and assessed these transcripts in our AS mice (Figure S4C) and human iPSC neuron (Figure S4D) samples. This again verified that the expression of the "sponge" *Ube3a1* is much lower than the (protein-coding) *Ube3a2/3* but is detectable. Taken together, these results indicate there is very limited brain region-specific dysregulation of miR-134 and its targets in AS mice, which may be due to the low expression of the *Ube3a1* sponge transcript.

AntimiR silencing of miR-134 in F1 *Ube3a*$^{m-/p+}$ mice alters open-field performance

We next investigated whether inhibiting miR-134 could alter key phenotypes in F1 *Ube3a*$^{m-/p+}$ mice. Silencing was achieved using a locked nucleic acid (LNA) antimiR targeting miR-134 (Ant-134),[32] (Figure 2E). F1 *Ube3a*$^{m-/p+}$ mice received a single intracerebroventricular (ICV) injection at P21 of 0.1 nmol Ant-134 or a scrambled control antimiR (Scr), and brains were extracted 24 h later, to coincide with maximal knockdown.[14] Ant-134 reduced levels of miR-134 in the hippocampus and cerebellum in F1 *Ube3a*$^{m-/p+}$ mice (Figures 2F and 2G).

Additional F1 *Ube3a*$^{m-/p+}$ mice were then injected with Ant-134 at P21 and studied at P22 for performance in a series of behavioral tests. Unexpectedly, 0.1 nmol Ant-134-treated F1 *Ube3a*$^{m-/p+}$ mice displayed increased exploration of the open field and higher entries into the middle zone compared with Scr-treated F1 *Ube3a*$^{m-/p+}$ mice (Figures 2H–2J; see Videos S1 and S2). Ant-134 did not affect distance traveled in the open field (Figure 2K) or improve performance of F1 *Ube3a*$^{m-/p+}$ mice in other assays, including the rotarod (Figure 2L) and marble-burying assay (Figure 2M). Re-testing the Ant-134 and Scr *Ube3a*$^{m-/p+}$ mice 3 weeks later revealed comparable performance in the open-field, rotarod, and nest-building assays (Figures S5A–S5F). Treatment of wild-type mice with Ant-134 did not affect performance in the open field, rotarod, or marble-burying assay (Figures S5G–S5P). Finally, we explored whether inhibition of miR-134 had effects on the resting EEG phenotype. Injection of Ant-134 into P42 F1 *Ube3a*$^{m-/p+}$ mice, an age when F1 *Ube3a*$^{m-/p+}$ mice displayed stronger EEG phenotypes, had no effect on resting EEG power (Figures S5Q and S5R) and Ant-134 mice displayed similar EEG frequency band distribution to Scr-injected *Ube3a*$^{m-/p+}$ mice (Figures S5S–S5V). This is consistent with previous reports in wild-type mice that silencing miR-134 does not alter resting-state EEG.[14]

Audiogenic seizure-susceptible *Ube3a*$^{m-/p+}$ mice

As F1 *Ube3a*$^{m-/p+}$ mice did not display an assayable epilepsy phenotype, we backcrossed onto a 129 background to generate mice susceptible to audiogenic seizures.[8] Backcrossing F1 *Ube3a*$^{tm1alb/J}$ mice to a 129Sv/Ev background for four generations generated N4 *Ube3a*$^{m-/p+}$ mice (Figure S6A). We confirmed that N4 *Ube3a*$^{m-/p+}$ mice displayed loss of Ube3a protein in the hippocampus, neocortex, and cerebellum (Figure S6B). We also analyzed the expression of miR-134 in the N4 generation. Levels of miR-134 and a selection of targets were not altered in the hippocampus or cerebellum (Figures S6D-S6J).

As expected, N4 *Ube3a*$^{m-/p+}$ mice responded to an audiogenic stimulus (repeated horizontal striking of a metal object across the top of the cage), undergoing short-lasting seizures that featured initial wild running, progressing to generalized tonic-clonic convulsions and then bilateral tonic extensions of the hindlimbs (Figures S6K–S6M).

AntimiR silencing of miR-134 in N4 *Ube3a*$^{m-/p+}$ mice reduces brain network excitability

We next confirmed that 0.1 nmol and a higher dose of 0.5 nmol of Ant-134 effectively inhibited miR-134 in N4 *Ube3a*$^{m-/p+}$ mice (Figures 3A and 3B). The knockdown of miR-134 by 0.5 nmol Ant-134 was associated with the de-repression of *Dcx* in the hippocampus (Figure 3C), but a selection of other miR-134 target levels did not change (Figures 3D and 3E).

Next, we investigated whether targeting miR-134 reduced brain hyperexcitability in the N4 *Ube3a*$^{m-/p+}$ mice. For this, we prepared *ex vivo* brain slices from N4 *Ube3a*$^{m-/p+}$ mice treated with Ant-134 at P21. We observed a specific reduction in the population synaptic potential in hippocampal CA1 (Figures 3F and 3H), likely indicative of reduced glutamatergic function in the network. The population spike, a measure of network action potential firing, was not changed by Ant-134 (Figures 3G and 3H). This suggests a relatively specific effect of Ant-134 to reduce hippocampal network excitability in juvenile N4 *Ube3a*$^{m-/p+}$ mice.

AntimiR silencing of miR-134 in N4 *Ube3a*$^{m-/p+}$ mice protects against audiogenic seizures

To explore the affect of inhibiting miR-134 on audiogenic seizures, P21 N4 *Ube3a*$^{m-/p+}$ mice received an ICV injection of Ant-134 or Scr, then 24 h later underwent a first audiogenic seizure (Figure 4A). To explore how long lasting these effects were, we elicited two additional audiogenic seizures, at 2 day intervals between seizures (Figure 4A).

On the first test day, Scr-treated N4 *Ube3a*$^{m-/p+}$ mice developed severe seizures, including tonic hindlimb extension. Ant-134 reduced audiogenic seizure severity in N4 *Ube3a*$^{m-/p+}$ mice compared with Scr-treated N4 *Ube3a*$^{m-/p+}$ mice (Figures 4B and 4C; see Videos S3 and S4). Specifically, two Ant-134-treated AS mice did not display any seizures upon audiogenic testing on the first test day, while the remaining Ant-134 mice displayed mainly lower severity seizures (Figures 4B and 4C). Ant-134-treated N4 *Ube3a*$^{m-/p+}$ mice remained protected against audiogenic seizures during the second and third tests (Figures 4B and 4C). There was also a tendency toward improved survival in Ant-134-treated N4 *Ube3a*$^{m-/p+}$ mice over the course of the audiogenic seizure testing (Figure 4D).

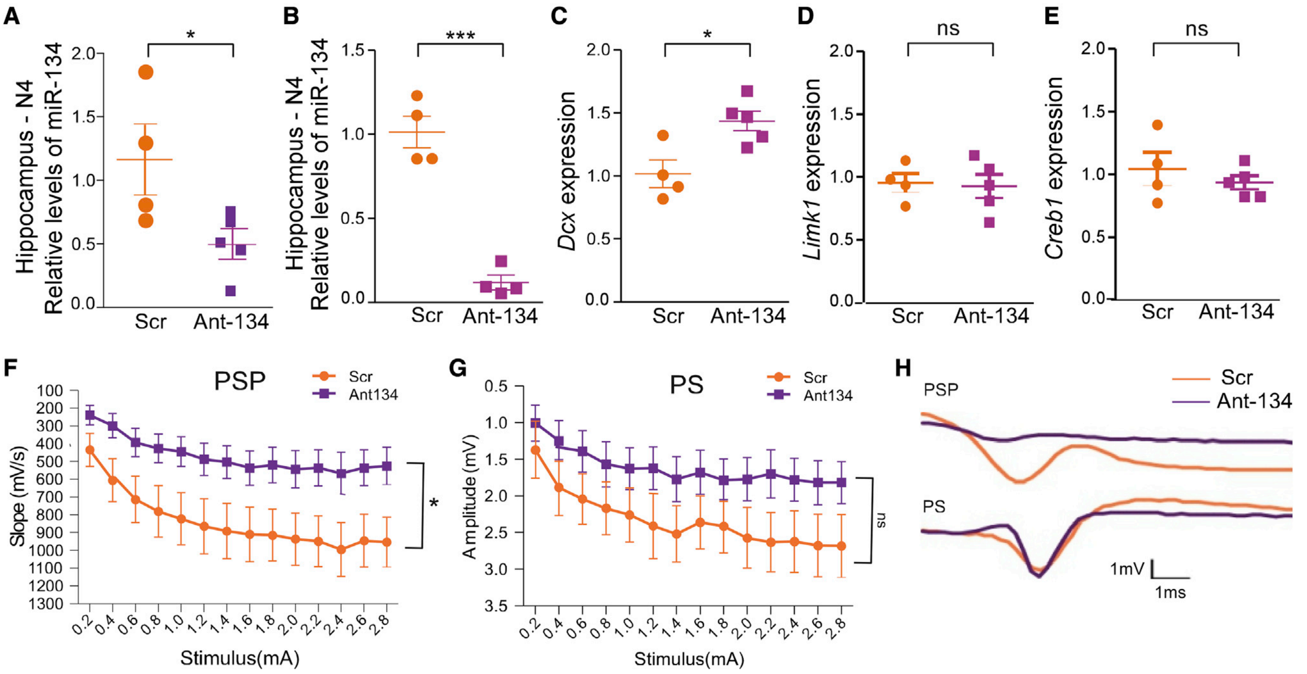

Figure 3. Silencing miR-134 reduces hippocampal excitability in N4 *Ube3a*$^{m-/p+}$ mice

(A and B) miR-134 levels in N4 generation *Ube3a*$^{m-/p+}$ P21 mice treated with Scr or Ant-134 with (A) 0.1 nmol or (B) 0.5 nmol; n = 4 or 5 group. Brains were analyzed 24 h later using qRT-PCR. *p = 0.042 and ***p = 0.0011. (C) *Dcx* transcript levels 24 h following Scr or Ant-134 (0.5 nmol); *p = 0.014. (D) *Limk1* transcript levels 24 h following Scr/Ant-134 (0.5 nmol). (E) *Creb1* transcript levels 24 h following Scr/Ant-134 (0.5 nmol); p = 0.90. (F) Brain slice recordings show Ant-134 caused a reduction in hippocampal CA1 population synaptic potential; n = 4 mice per group; 2 or 3 slices per mouse; two-way repeated-measures ANOVA, treatment term *p = 0.038. (G) Ant-134 had no effect on hippocampal CA1 population spiking; n = 4 mice per group; 2 or 3 slices per mouse; two-way repeated-measures ANOVA, treatment term p = 0.15. (H) Representative raw traces showing population synaptic potential and spiking in hippocampal CA1 in Ant-134 and Scr-treated *Ube3a*$^{m-/p+}$ mice. Data are expressed as SEM. Multiple corrections were performed on molecular data, and α was adjusted to 0.016. ns, non-significant.

To support a specific effect of miR-134 inhibition on the observed anti-seizure effects, brains were obtained 24 h after the final seizure (at P27). Although Limk1 levels were not significantly different, protein levels of miR-134 targets Dcx and Creb1 were both increased in Ant-134-treated N4 *Ube3a*$^{m-/p+}$ mice compared with Scr at the end of audiogenic seizure testing (Figures 4E–4G).

We further explored the duration of action of Ant-134, repeating the triple audiogenic seizure tests but spaced over a longer time period. The first seizure was given the day after Ant-134 treatment (P22), followed by re-testing of AS mice with a second audiogenic seizure at P28 and a final test at P42. Results of the first audiogenic test were in line with our previous observations with Ant-134 protecting against seizures (Figure S7). However, the anti-seizure effects of Ant-134 were less obvious over subsequent test weeks, and the effect did not reach significance (Figure S7).

We then investigated whether the anti-seizure effects of Ant-134 would extend to older AS mice. P42 N4 *Ube3a*$^{m-/p+}$ mice were given either Ant-134 or Scr prior to triple audiogenic seizure testing over subsequent days, separated by 2 day intervals (Figure 4H). Notably, seizure severity was lower in Scr-treated N4 *Ube3a*$^{m-/p+}$ mice at this age, as reported.[33,34] Nevertheless, Ant-134 significantly reduced seizures in P42 N4 *Ube3a*$^{m-/p+}$ mice over the course of three test audiogenic stimuli compared with Scr-treated AS mice (Figures 4I and 4J). Taken together, these results indicate silencing miR-134 has anti-seizure effects in AS mice and this extends into adulthood.

Gene expression profile after Ant-134 treatment

To obtain insights into potential mechanisms by which Ant-134 produces anti-seizure effects in AS mice, we performed RNA sequencing (RNA-seq) analysis of hippocampal samples from N4 *Ube3a*$^{m-/p+}$ mice treated with 0.5 nmol Ant-134 in comparison with the Scr-treated littermates. This identified 1,027 differentially expressed genes (unadjusted p value), of which 405 were upregulated and 622 were downregulated in the Ant-134-treated mice in comparison with the Scr group (Figure S8A). We next focused on upregulated genes that were (1) predicted miR-134 targets previously identified as differentially expressed after Ant-134 treatment[28] or (2) associated with epilepsy (Table S1). This identified 52 upregulated genes. These were significantly enriched for seven Reactome pathways, namely, *Activation of Ca-permeable kainate receptor*, *ionotropic activity of kainate receptors*, *transmission across*

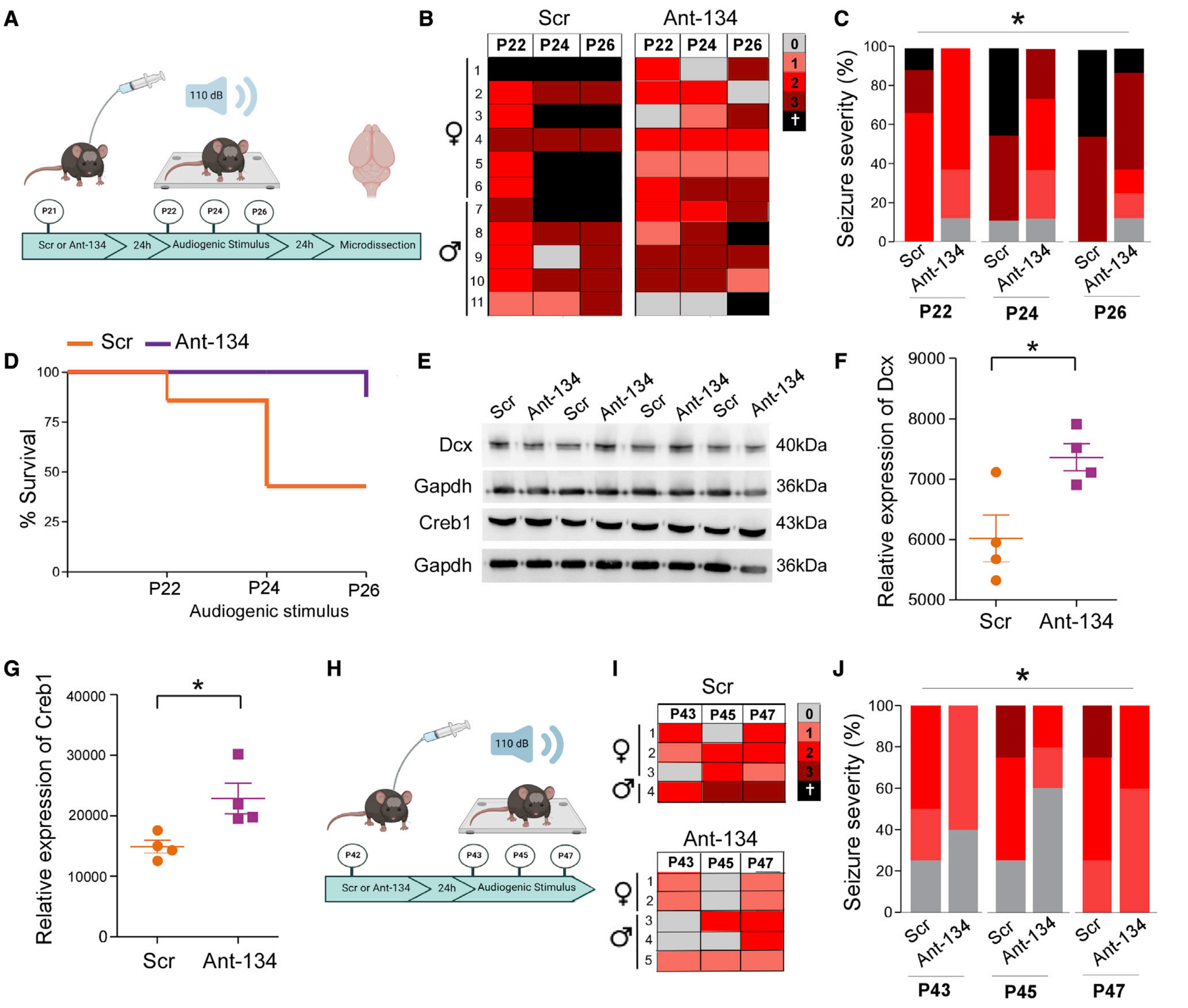

Figure 4. Ant-134 reduces audiogenic seizures in N4 *Ube3a*$^{m-/p+}$ mice

(A) Schematic of antimiR testing in the audiogenic seizure model. P21 *Ube3a*$^{m-/p+}$ mice received an ICV injection of Scr/Ant-134 (0.5 nmol), and seizures were induced 24 h later. Seizures were repeated at P24 and P26. (B) Tabular representation of the type of seizure each mouse had over three seizures. (C) Percentage of maximum seizure score per treatment group at P22, P24, and P26. There was a significant reduction in seizure severity in Ant-134 treated mice at P26 in comparison with P22; Scr, n = 11; Ant-134, n = 11; *p < 0.05. (D) Kaplan-Meier curve of survival rates following Scr/Ant-134. Note, 55% survival in Scr group in comparison with 81% survival in Ant-134 group (ns). (E) Representative western blots show levels of Dcx 24 h after final seizure in the cortex and Creb1 levels in the hippocampus of Scr/Ant-134-treated mice. (F) Dcx in the cortex; n = 4/genotype, *p = 0.024. (G) Creb1 levels in the hippocampus; n = 4/genotype, *p = 0.026. Data are expressed as SEM. (H) Schematic illustration of experimental design of antimiR testing in P42 AS mice. (I) Tabular representation of the type of seizure each mouse had over three seizures. (J) Percentage of maximum Racine score per treatment group at P22, P24, and P26; Scr, n = 4; Ant-134, n = 5; *p = 0.04. Mortality was compared between treatments using incidence rate ratios and a Poisson model, with number of trials as the exposure variable. Seizure severity was modeled using ordinal logistic regression with robust variance estimation used to adjust for clustering of data with mice. An interaction term was used to test for a change in effectiveness of treatment as a function of trial number.

chemical synapses, *HSF1 activation*, *calcineurin activates NFAT*, *FCERI mediated Ca*$^{2+}$ *mobilization*, and *CLEC7A (Dectin-1) induces NFAT activation* (Figures S8B and S8C). Thus, RNA-seq analysis indicates antimiR-134 produces effects on a large set of genes in AS mice, including direct as well as indirect targets with links to control of neuronal excitability and epilepsy.

Ant-134 effects in human neurons from AS patient-derived iPSCs

Finally, we sought proof of concept that this antimiR approach can produce effects in a human model. We differentiated a mixture of excitatory and inhibitory neurons from induced pluripotent stem cells (iPSCs) obtained from a female AS patient with a

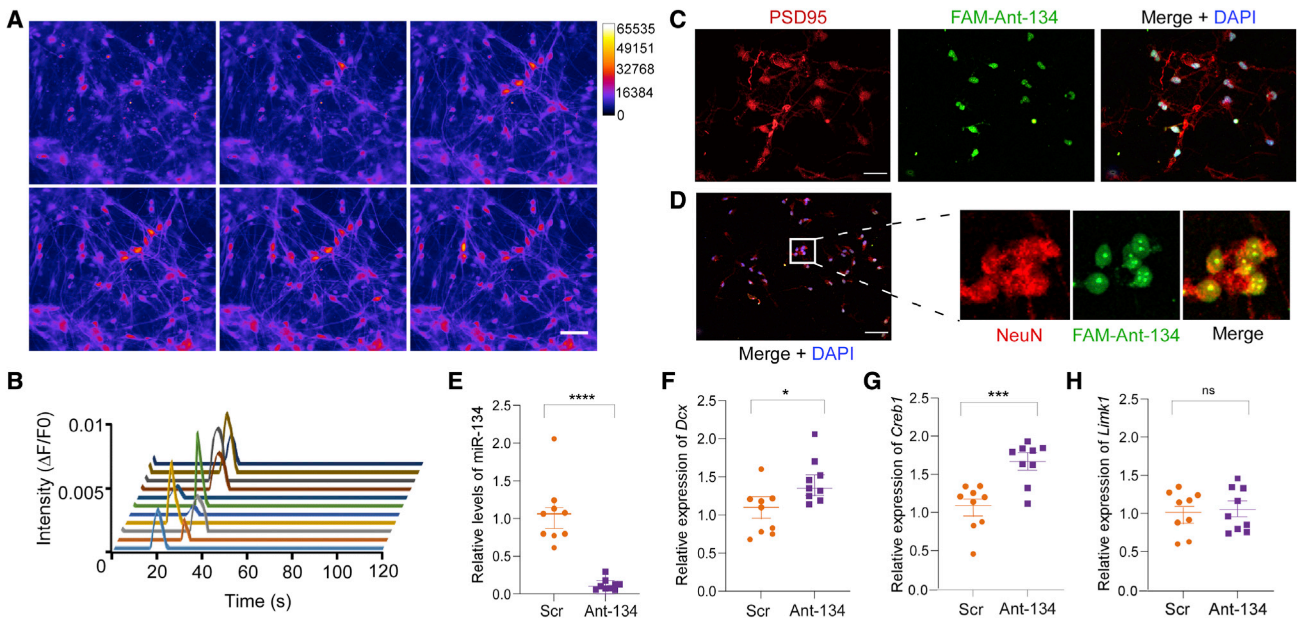

Figure 5. Ant-134 reduces miR-134 levels and upregulated targets in AS patient-derived neurons

(A and B) Recorded calcium transients in differentiated neurons (6 weeks) from an AS patient showing typical spontaneous activity consistent with functioning network. (C and D) Detection of Ant-134 (fluorescein amidite [FAM]-labeled) 24 h after transfection in neurons expressing PSD-95 and NeuN. (E) Potent knockdown of miR-134 by Ant-134 (300 nM) after 24 h treatment in 6 week-differentiated AS patient neurons. (F–I) Transcript levels of miR-134 targets in Ant-134-treated AS neurons. Note increased levels some (*DCX*, *CREB1*) but not all (*LIMK1*, *Serpine1*). n = 9 Ant-134, n = 9 Scr; *$p < 0.05$, **$p < 0.01$, and ***$p < 0.001$. Data are expressed as SEM. Multiple corrections were performed on molecular data, and α was adjusted to 0.01. ns, non-significant.

15q11.2-q31.1 deletion and confirmed epilepsy.[35] Differentiated neurons at 6 weeks displayed appropriate morphological features and markers of network maturity, including spontaneous calcium transients (Figures 5A and 5B; Video S5). Transfection of neurons for 24 h with a fluorophore-labeled version of Ant-134 confirmed uptake in postsynaptic density protein-95 (PSD-95)-positive cells and cells expressing the mature neuronal marker NeuN (Rbfox3) (Figures 5C and 5D). We then treated additional cultures of AS neurons with either unlabeled Ant-134 or Scr (300 nM) for 24 h and extracted RNA for analysis of miR-134 and targets. Levels of miR-134 were potently suppressed in Ant-134-treated neurons compared with Scr (Figure 5E). Transcript levels of *DCX* and *CREB1* but not *LIMK1*, were upregulated in Ant-134-treated neurons (Figures 5F–5H).

DISCUSSION

The molecular mechanism underlying AS lends itself to treatment approaches that re-establish the normally silenced paternal copy of *UBE3A*.[1] Limitations have emerged, however, in the effectiveness of gene re-reinstatement to reverse various phenotypes, particularly in older mice,[5-9] necessitating exploration of other approaches. Here we explored a potential molecular defect predicted to arise in AS because of the loss of miRNA binding sites, or sponge function, of the *Ube3a1* transcript.[11] *Ube3a1* has been reported to contain binding sites for multiple miRNAs from the miR-379∼410 cluster including miR-134.[36] Loss of *Ube3a1* has been reported to alter dendritic spine size and excitatory currents in neurons.[11] There is a pre-existing association of miR-134 with fine-tuning brain excitability,[19,37] and targeting miR-134 is effective at reducing evoked and spontaneous seizures in models of epilepsy.[38] Drug-resistant epilepsy is a serious problem in AS, and seizure control is both important and inadequate for a majority of patients.[1,2]

We found that a single central injection of antimiRs against miR-134 was able to attenuate the epilepsy phenotype in audiogenic seizure-susceptible AS mice. Ant-134 was effective in both P21 and older (P42) AS mice, and seizure severity remained reduced for several days afterward. Indeed, although audiogenic seizure severity increased in the model over repeated test days, likely because of a kindling or sensitization effect, seizure suppression by Ant-134 sustained. Treating the epilepsy phenotype in AS mouse models has often been incomplete or ineffective using targeted reinstatement of *Ube3a*, particular in older animals.[8,9] Targeting miR-134 does not appear to be as age restricted, offering a possible advantage over current gene therapy rescue approaches. The extent of seizure suppression is similar to effects of Ant-134 in chemoconvulsant models,[14,16,23] and compares well with or is superior to pharmacologic and genetic approaches to hyperexcitability that focus on restoring *Ube3a*.[5,8,9]

The anti-seizure mechanism of Ant-134 is incompletely understood but may involve reducing glutamatergic input. Here, we showed

potential involvement of neuronal and Ca^{2+}-related pathways that may modulate synaptic transmission, plasticity, synaptogenesis, or synaptic pruning. An effect via dendritic spines is possible, as silencing miR-134 adjusts spine number and volume in excitatory hippocampal neurons,[16,19] and the anti-seizure and neuroprotective effects of Ant-134 in other models are diminished by suppression of *Limk1*.[14,28] Levels of *Limk1* were not, however, increased by Ant-134 in AS mice or AS patient-derived neurons and the human 3′ UTR of *LIMK1* lacks the canonical seed for miR-134.[14,19,39] We observed that inhibition of miR-134 reduced the population synaptic potential in area CA1 of the hippocampus of AS mice while largely sparing the population spike. This is consistent with a reduction in glutamatergic transmission but not the properties of single action potentials. This effect may be mediated via changes to other transcripts altered by Ant-134 in the mouse hippocampus.[28] It is possible that reducing miR-134 in other brain regions, including the cerebellum, was involved in the seizure suppression. Indeed, pathways from the cerebellum are increasingly recognized as capable of influencing seizures.[40]

The LNA-modified antimiRs we used to target miR-134 produce potent, specific, and lasting miRNA knockdown.[14] The same chemistry was used to target other, non-CNS miRNAs that reached clinical trials previously.[41] LNA-modified antimiRs can suppress their targets for many weeks following a single injection, although maximal knockdown after ICV injection of Ant-134 peaks during the first week.[14] We found that the anti-seizure effects of Ant-134 were no longer detectable when AS mice were re-tested 1 and 3 weeks later. This suggests that seizure suppression using this approach for AS may require high or sustained silencing of miR-134. This contrasts with the multi-week effectiveness of a single injection of Ant-134 on epileptogenesis in models of status epilepticus.[14,24,28] This may be because there is an enduring genetic mechanism in AS. However, optimizing dosing and delivery routes may enhance or extend the duration of effect.[14,28] What would be the prospects for a clinical trial using a miRNA-based treatment approach? AntimiRs would require a delivery approach that circumvents the blood-brain barrier such as intrathecal injection.[42] Such invasive delivery methods are acceptable if balanced against disease severity. Indeed, clinical trials of UBE3A-rescuing strategies are under way for AS that use intrathecal delivery of ASOs.[43] An antimiR-based miRNA inhibitor would have similar pharmacokinetic and safety challenges, albeit with possibility of small, widespread effects on gene expression due to the multi-targeting action of miRNAs. There are alternative methods for silencing miRNAs, such as using gene therapy-based delivery of targeting sequences, which could offer longer lasting or cell type-specific delivery.[44]

An interesting and unexpected finding here was that silencing miR-134 altered performance of AS mice in the open field. Movement and anxiety defects are a consistent phenotype in AS mice, and we observed impaired ambulatory performance in open-field exploration and rotarod. There was a significant increase in activity levels in Ant-134-treated mice, with animals spending more time exploring the open-field arena compared with Scr-treated AS mice, although Ant-134 did not improve rotarod performance. The group sizes we used are lower than recently recommended,[26] although they are in line with other work.[34] Notably, although motor incoordination can be partly rescued by re-expression of *Ube3a* in juvenile mice, the open-field defect cannot.[7,8] A leading alternative therapeutic strategy targeting insulin-like growth factor (IGF) also fails to correct open field deficits.[10] This further supports the targeting of miR-134 to alleviate certain phenotypes in AS, either alone or in combination with *Ube3a* reinstatement approaches, particularly at older ages. The open-field test is a general measure of exploratory behavior and activity, and the circuit basis includes elements of motor, navigational, and anxiety features. Anxiolytic drugs produce similar improvements to those we observed with Ant-134.[45] However, multiple factors affect performance of mice and we must be careful not to ascribe the effects of Ant-134 to reducing, for example, anxiety. Indeed, an alternative interpretation of our finding would be that treatment with the anti-miR results in a loss of cautionary avoidance of a novel open area.[46] The mechanism by which targeting miR-134 improves open-field performance is unknown. Notably, Ant-134 increased levels of Creb1 in the hippocampus and Dcx in the neocortex of audiogenic seizure-exposed AS mice and levels of *CREB1* and *DCX* increased in AS patient-derived neurons treated with Ant-134. Although manipulation of Dcx is not associated with phenotypes in this assay,[47] an effect on Creb1 could potentially account for the observations.[48] It is possible that effects on other targets are important,[28] and the neurobiological basis of the various phenotypes corrected or not by Ant-134 requires further study. Effects of Ant-134 within the cerebellum are also possible, as lesions and abnormalities of the cerebellum are associated with decreased exploration of novel environments such as the open field in rodents and humans.[49,50]

Elevated levels of miR-134 were detected in the cerebellum of F1 AS mice, but miR-134/target expression appeared otherwise normal in AS mice. Thus, the loss of *Ube3a1* is insufficient to disturb expression of either miR-134 or its targets. This may be because of the low expression of *Ube3a1*/*UBE3A1* found here, and reported by others,[51] which may limit its functional relevance. Technical factors should also be considered. Our assay methods may lack sensitivity to detect small or temporally variable changes in miR-134 or its targets, and cell type-specific dysregulation of miR-134 would have been masked in whole-tissue analyses. Most likely, the protective effects of Ant-134 occur independently of the regional molecular landscape adjustment upon targeting miR-134. Notably, anti-seizure effects of Ant-134 have been reported in models when miR-134 was not elevated.[23] Further studies are needed to look at the broader miRNA landscape in AS, including other miRNAs predicted to be sequestered by *Ube3a1* and to better elucidate the relationship between *Ube3a1* and miR-134 in specific brain cell types in AS.

The present study identified limitations in the efficacy of targeting miR-134 in AS. Ant-134 failed to improve several phenotypes, including rotarod performance, which can be partly rescued by *Ube3a* reinstatement[6,8] or promoting IGF signaling.[10] The duration of anti-seizure effects of Ant-134 is in contrast with the long-lasting attenuation of seizures in rodent models of acquired, focal epilepsy.[28]

If a miRNA-based strategy is to be developed for targeting AS, it might be necessary to combine this with *Ube3a* reinstatement or potentially target additional miRNAs. Future studies could explore the full miRNA landscape to identify additional targetable miRNAs for AS. Notably, the miR-379∼410 cluster of which miR-134 belongs contains miRNAs that control brain development, synapse structure and excitability,[36,52] and miRNAs linked to anxiety,[53] and sociability,[54] which are other, largely untreatable aspects of the clinical spectrum of AS. This might necessitate a combinatorial approach, as demonstrated recently using a pool of antimiRs to treat a set of upregulated miRNAs in mice with drug-resistant epilepsy.[17] Another potentially relevant miRNA for targeting is miR-211, located within the Chr 15q.13.3 locus, deletions of which are associated with severe epilepsy and intellectual disability. Overexpression of miR-211 disrupts multiple synapse-related pathways, interferes with learning and memory and evokes seizures in mice, suggesting its antimiR targeting of miR-211 could be an alternative strategy in AS.[55] It is possible that antimiR targeting of miR-134 could have applications in other imprinting or neurogenetic disorders. Besides the anti-seizure effects reported here, which are probably independent of a miRNA sponge effect of *Ube3a1*, inhibition of miR-134 has been reported to restore cognitive functions in mice with learning and memory deficits.[20] There are more than 200 imprinted genes in humans, and a number feature cognitive and psychiatric features as well as epilepsy.[56] Notably, motor and cognitive deficits as well as seizures are common in patients with duplication of 15q11-q13, which harbors a number of imprinted genes and is associated with increased UBE3A levels. Prader-Willi syndrome (PWS) is considered the reciprocal imprinting disorder of AS, where a paternally expressed cluster of contiguous genes on chromosome 15 are lost. But epilepsy is not part of the normal clinical spectrum of PWS. If *LIMK1* is a target of miR-134 in humans, and this remains unclear, there might be an application in Williams-Beuren syndrome (WBS), the clinical features of which include locomotor problems and anxiety, although epilepsy is rare.[57] *LIMK1* is one of the genes commonly deleted in WBS, potentially resulting in increased miR-134 function. More broadly, miRNA targeting could offer ways to correct the effects of duplication of individual or miRNA cluster-related disorders.[58]

In summary, the present study reveals a potential precision therapy approach based on targeting a miRNA associated with the molecular mechanism of AS. We show that antimiR ASOs are effective at reducing miR-134 in AS mice and produce seizure suppression and improvements in open-field performance. Anti-seizure effects spanned juvenile and older mice, indicating a protracted developmental window for this approach. Further studies will be needed to optimize this strategy and explore whether combinations, either with silencing of other miRNAs or approaches directly focused on re-expression of the *Ube3a* gene.

MATERIALS AND METHODS

Animals

All animal experiments were reviewed and approved by the Research Ethics Committee of the Royal College of Surgeons in Ireland (REC 1302bbb) under licenses from the Health Products Regulatory Authority (AE19127/P013 and AE19127/P064), in accordance with the European Communities Council Directive (2010/63/EU). Animal work was performed by either A.C. (AE19127/I178) or C.R.R. (AE19127/I018). Male C57BL/6 mice (P7-42) were obtained from RCSI's Biomedical Research Facility (original stock from Harlan, Oxon, Bicester, UK). P7 and P14 mice were housed with littermates and dam until non-recovery experiments. *Ube3a* mutant mice were obtained from Jackson Laboratory (Bar Harbor, ME; stock no. 016590) and bred as detailed below. Weaned animals were housed (up to 5 mice per cage) in on-site barrier-controlled facilities with a 12 h light-dark cycle and *ad libitum* access food and water.

Ube3a $^{(m-/p+)}$ mice

The AS mice colony was initiated using B6.129S7-*Ube3a*^{tm1alb}/J mice carrying a mutation in *Ube3a* maintained on C57BL/6 background. *Ube3a* $^{(m-/p+)}$ adult males were crossed with wild-type C57BL/6 females from the maintenance colony. Heterozygous (het) females generated from the maintenance colony were crossed with wild-type 129Sv/Ev males from the maintenance colony to generate the experimental colony. The maintenance colony was bred continuously to generate wild-type 129Sv/Ev males. Mice generated from this colony were from the first generation. To generate mice that were susceptible to audiogenic seizures, F1 mice were backcrossed for four generations and maintained on a 129Sv/Ev background until N3 female heterozygotes were available to breed with males from the 129Sv/Ev maintenance colony. AS mice of both sexes were used for behavior and EEG/seizure studies but ratios of male:female were not recorded for molecular studies. All molecular analysis was performed using tissue obtained from saline-perfused mice.

Behavioral trials

All behavioral experiments were performed between 1 and 6 p.m. Mice in their home cages were placed in the behavior room 30 min prior to testing to habituate. Apparatus was cleaned with 30% ethanol before use and between trials. Similar numbers of male and female wild-type and AS mice were used in all behavioral tests.

Open field

Locomotor activity was tested using the open-field test. Wild-type and AS mice were placed individually in the middle of a test chamber (27.3 × 27.3 cm; Med Associates, Inc., Fairfax, VT) and allowed to explore the arena for 10 min. Movement was recorded using a Logitech HD 1080p camera. Data were analyzed by a researcher blind to genotype/treatment by manually recording the length of time the mouse was active during the trial. Next, using the AnyMaze (version 6.1) software (Stoelting, Dublin, Ireland), the exact dimensions of the arena were identified on the video by a ruler tool (30 mm). The inner and outer zones of the arena were also identified to track the number of crossings between zones and the total time spent in each area. The mouse's head and tail were identified in the software and tracked throughout the trial. The following parameters were measured: total distance traveled (mm), speed (m/s), entries into inner zone, and total

time spent in inner zone (s). Exploration time was analyzed manually by recording the length of time the mouse was active during the 10 min trial. Active refers to the mouse being mobile using all four limbs in the arena.

Accelerating rotarod

Motor function was assessed using the accelerating rotarod (Letica Scientific Instruments, Portugal). A trial period was first performed in which mice were placed on the rotarod for 3 min at a constant speed. Mice were placed back onto the rotarod apparatus if they fell off during the trial. During the test phase, mice were placed onto the rotarod at an accelerating speed of 4–40 rpm, for 5 min. This trial and testing phase were repeated on two consecutive days at the same time of the day, with two trials per day with at least 40 min between each test. The latency to fall (s) per mouse was recorded, and the average of two trials was taken.

Light-dark box

Ube3a$^{m-/p+}$ and wild-type mice were individually placed in the open-field chamber (Med Associates, Inc.) for 10 min. A dark-box insert (Med Associates, Inc.) was placed into the chamber and represented the dark section of the arena. A light (60 W/600 lux) was used to illuminate the light section of the arena. Upon trial initiation, the mouse was placed into the light compartment. The time spent in each compartment was analyzed manually and using AnyMaze software, as before. A full transition was identified and recorded when all four paws were in the given arena.

Marble burying

Abnormal behaviors and autistic like features were assessed using the marble-burying assay. Polycarbonate cages (365 × 207 × 140 mm) were filled with 5 cm of bedding. Twenty marbles were placed on top of the bedding and arranged in 5 × 4 grid at equal distance. Mice were placed into the middle of the cage for a 30 min period. After the trial, the amount of marbles with half or more of their surface buried in the bedding were counted.

Nest building

The nest-building assay was used to test the naturalistic behaviors. Before commencing the nest building test, mice were single-housed for a week. Twenty-four hours prior to testing, environmental enrichment was removed from each cage. On the first day of testing, 10 g of blotting filter paper (Bio-Rad) was placed in each cage. The amount of unused nesting material was weighed daily, at the same time of the day, for 5 consecutive days. For statistical analysis, the average weight of blotting paper was graphed.

Electrode implantation and ICV injections

Mice were anesthetized with isoflurane (5% induction, 1%–2% maintenance) and placed in a mouse-adapted stereotaxic frame. After local analgesia with lidocaine and prilocaine cream (EMLA), a midline scalp incision was made, bregma was located, and three partial craniotomies were performed (two cortical electrodes placed behind bregma and the reference electrode placed toward the front of the brain, above bregma on the right-hand side). Mice received 2 μL mmu-miR-134-5p miRCURY LNA power inhibitor (Ant-134; 5′-3′ tggtcaaccagtcaca/3CholTEG Exiqon; 0.1 or 0.5 nmol in PBS), a non-targeting scrambled control (Scr, 5′-3′ cgtctagccacctag/3Chol-TEG), or vehicle (PBS), injected using a Hamilton syringe at a rate of 1 μL/min. The electrode assembly was fixed in place using dental cement. After surgery, the mouse was placed in a temperature-controlled open Perspex box and monitored.

Seizure induction

To evaluate sensitivity to seizures, F1 mice were given a systemic (i.p.) injection of a subconvulsant dose of pentylenetetrazole (PTZ; 40 mg/kg) or kainic acid (KA; 10 mg/kg) (both from Sigma-Aldrich). LabChart software was used to assess EEG changes including total power, amplitude, and frequency of any ictal activity.

For audiogenic seizures, we first tested an alarm-based method in F1 mice implanted with surface EEG electrodes. In pilot tests, mice were individually placed in a soundproof chamber (SR-Lab, Startle response system, San Diego Instruments), and tethered swivel cables were wired through the back of the chamber for EEG recordings. A Logitech HD 1080p camera was placed inside the chamber. An alarm (Screaming Meanie 220; Pacific Conrnetta, Oregon) was set to desired time and decibel level (110–140 dB) and also placed inside the chamber. Mice were left for 15 min to habituate to the chamber and to record baseline EEG. The alarm went off at the desired time and if repeated, with 10 min between each audiogenic stimulus. For N4 generation AS mice, a different model was used. P21 and adult mice were placed in a polycarbonate cage (365 × 207 × 140 mm), and the lid of the cage was vigorously scratched for 30 s (generating approximately 110 dB). The seizures were recorded by a Logitech HD 1080p camera and analyzed using an adapted audiogenic seizure scale. The maximum score per seizure type was noted per mouse, according to an audiogenic seizure scale.[59] The latency to wild running (type 1), generalized tonic-clonic convulsions (type 2), and tonic hyperextension of the hindlimbs and tail (type 3) was identified by video analysis This method of seizure induction was unsuccessful in F1 mice.

Argonaute-2 immunoprecipitation

Hippocampus was homogenized by hand in 500 μL immunoprecipitation buffer (150 mM NaCl, 20 mM Tris-HCL [pH 7.5], 5 mM $MgCl_2$, and 1% NP-40) and centrifuged. Lysate (500 μL) was added to agarose beads and incubated for 2–6 h on a rotator at 4°C. Samples were incubated overnight at 4°C with 10 μg Ago2 antibody (1:10, C34C6; Cell Signaling Technology). The lysate-bound antibody solution was added to agarose beads and left on the rotator for 2 h. The supernatant was removed and the pellet was suspended in 200 μL TRIzol, and an RNA extraction was performed.

Standard RNA extraction

Brain samples were homogenized in 750 μL TRIzol (re-suspended in 200 μL for synaptoneurosomes and AgoIP) and centrifuged at

12,000 × *g* for 10 min at 4°C. Phase separation was performed by adding 200 μL chloroform to each sample (75 μL for synaptoneurosomes and AgoIP) and vigorously mixing for 15 s before incubating at room temperature (RT). Samples were centrifuged at 15,600 × *g* for 15 min at 4°C. The upper phase was removed and 450 μL isopropanol was added, and samples were stored at −20°C overnight. Samples were centrifuged at 15,600 × *g* for 15 min at 4°C. Seventy-five percent cold ethanol (750 μL [200 μL for AgoIP]) was used to wash the pellet. Samples were centrifuged at 13,300 × *g* for 5 min, and the ethanol was removed. The pellets were left to dry for 1 h and re-suspended in 25 μL (8 μL for AgoIP) RNase-free H_2O. Samples were incubated for 10 min at 60°C with 60 × *g* agitation.

miRNA expression

miRNA (500 ng) was reverse-transcribed using stem-loop Multiplex primer pools (Applied Biosystems, Dublin, Ireland). We used reverse transcriptase-specific primers for the mmu-miR-134 (Applied Biosystems miRNA assay ID 00186), and real-time quantitative PCR was carried out on a 7900HT Fast Realtime System (Applied Biosystems) using TaqMan miRNA assays (Applied Biosystems). U6B (Applied Biosystems miRNA assay ID 001093) was used for normalization. A relative fold change in expression of the target gene transcript was determined using the comparative cycle threshold method ($2-^{\Delta\Delta}$CT).

RNA sequencing

RNA-seq was performed by Novogene to analyze differential gene expression in mice hippocampi after Ant-134 treatment (n = 5; 0.5 nmol) in comparison with Scr control (n = 4) after the repetitive audiogenic stimulus. mRNA was purified using poly-A selection, and sequencing libraries were prepared. Libraries were sequenced on an Illumina platform, generating an average of 46.9 million 2 × 150 bp paired-end reads per sample. Low quality and adapter-containing reads were removed, and clean reads were mapped to the GRCm38 reference genome using Hisat2 version 2.0.5. Reads mapped to each gene were counted using featureCounts version 1.5.0-p3, and differential expression between treatment groups was performed using DESeq2. Genes with p values < 0.05 and $|\log_2$ fold change$| > 0$ were considered differentially expressed. In order to identify the most relevant differentially expressed genes (DEGs), we filtered the DEG list using the following criteria: upregulated after Ant-134 treatment in AS mice, being a predicted miR-134 target according to the miRDB database,[60] having previously been identified as differentially expressed after Ant-134 treatment,[28] or being associated with epilepsy by cross-referencing and in-house curated database,[17] and by mining from OMIM publication searchers (summarized in Table S1). Finally, Reactome pathway enrichment analysis was performed on the DEGs of interest; pathways with adjusted p values < 0.05 were considered significantly enriched. RNA sequencing data are available from Gene Expression Omnibus (GEO) submission number: GSE199276.

Western blotting

Protein was extracted from brain samples, separated using SDS-PAGE, transferred to nitrocellulose membranes, and incubated in the following primary antibodies: Limk1 (1:500; Cell Signalling-3842), Creb1 (1:50; Santa Cruz-271), DCX (1:500; Cell Signalling-4604S). Membranes were then incubated in secondary antibodies, and bands were visualized using Supersignal West Pico Chemiluminescence Substrates (Pierce) and captured using a FUJIFILM Las-4000. Thereafter, membranes were stripped (200 mM glycine, 0.1% SDS, 1% Tween 20; pH 2.2), blocked, and incubated with GAPDH antibody (1:1000, Thermo Fisher Diagnostics AM4300), secondary antibody and visualized as above. Densitometry was performed using ImageJ software.

Ex vivo electrophysiology

P21 N4 male and female *Ube3a*$^{+/-}$ mice were injected with ICV Ant-134 or scramble control, as above. After 2–4 days, mice were euthanized by cervical dislocation and their brains were quickly dissected and submerged in oxygenated ice-cold sucrose artificial cerebrospinal fluid (composition: 205 mM sucrose, 10 mM glucose, 26 mM $NaHCO_3$, 1.2 mM $NaH_2PO_4.H_2O$, 2.5 mM KCl, 5 mM $MgCl_2$, 0.1 mM $CaCl_2$). Horizontal slices (400 μm) were prepared using a vibratome (Campden 7000 smz II; Campden Instruments, Loughborough, UK), with bath temperature held at ∼1°C. Slices were stored at room temperature in a submerged-style holding chamber, filled with oxygenated recording ACSF (125 mM NaCl, 10 mM glucose, 26 mM $NaHCO_3$, 1.25 mM $NaH_2PO_4.H_2O$, 3 mM KCl, 2 mM $CaCl_2$, 1 mM $MgCl_2$). For recording, slices were transferred to a membrane chamber that was perfused with oxygenated ACSF and heated to 34°C, at a rate of 16 mL/min.[61,62] Slices were equilibrated to these conditions for 1 h prior to recording. A stimulating electrode (SS3CEA4-200; MicroProbes, Maryland) was placed in to the Schaffer collateral pathway and 10 ms current pulses were delivered using a DS3 isolated current stimulator (Digitimer; Welwyn, Garden City, UK). Extracellular borosilicate glass recording electrodes (∼3 MΩ) were filled with ACSF and placed in stratum radiatum (SR) and stratum pyramidale (SP) in hippocampal CA1. Signal was acquired using a MultiClamp 700B amplifier (Molecular Devices, California), Power 1401 digitizer (Cambridge Electronic Design, Cambridge, UK), and Signal software (version 6; CED). Signals were digitized at 25 kHz and low-pass-filtered at 10 kHz. Data were analyzed using Signal version 6. Population spike was measured as the maximum trough amplitude recorded from CA1 SP and population synaptic potential as the slope of the response recorded in CA1 SR.

iPSC-derived Angelman neurons

Generation of cells was approved by Boston Children's Hospital (Boston, MA) institutional review board (IRB) (P00000219). Angelman patient-derived iPSCs (CRA_1501) were generated as recently reported.[35] Briefly, blood cells were obtained from a female patient, 11 years of age, with a 15q11.2-q13.1, 4.973 MB deletion, diagnosed with AS and with neurological features including epilepsy, lack of speech, and sleep disorder. Reprogramming was performed using Sendai virus-based method at the Harvard Stem Cell Institute. Neuronal precursors were grown in neuronal differentiation medium (DMEN/F12, Neurobasal media B27 supplement and N2

supplement and β-mercaptoethanol) with growth factors (EGF, FGF, BDNF, SHH (sonic hedgehog), FGF8, AA (Aa2-P), Dibutyral-cAMP-Na salt, and GDNF). Cells were plated on 6-well plates and coverslips coated with BioLaminin (5 μg/mL) at a density of 45,000 cells/cm^2 and maintained in an incubator under controlled temperature and CO_2 conditions (37°C, 5% CO_2) for 21–41 days. Neurons were treated with either a FAM-labeled (300 nM) or PBS for imaging, and unlabeled Ant-134 (300 nM) or Scr (300 nM) for RNA experiments for 24 h.

For imaging of spontaneous network activity, iPSC-derived neurons differentiated on glass coverslips were loaded with the calcium indicator Fluo-4 AM (2 μM, Molecular Probes, USA) in culture medium for 40 min at 37°C. Live calcium imaging was performed in imaging buffer using a Zeiss Axio Examiner microscope with a 40× water immersion objective (Zeiss W Plan-Apochromat 40×/1.0) and images were acquired at 1 Hz. Images were analyzed using Fiji software.[63] Fluo-4 traces were generated by averaging pixel intensity within the regions of interest (ROIs), and normalized on the baseline fluorescence of the first 6 frames (ΔF/F0).

For immunohistochemistry, differentiated cells on coverslips were fixed with 4% PFA in PBS, permeabilized with 0.1% Triton X-100, blocked with 1% FBS, and incubated with primary antibodies against NeuN (Merck MAB377) and PSD-95 (Abcam, Ab18258) for 1 h, followed by fluorophore-labeled secondary antibodies and DAPI counterstained for 45 min. Cells were washed again, mounted, and imaged using a fluorescence microscope (Leica DM4000b). RNA extraction was performed using the TRIzol method. For miRNA analysis, 250 ng RNA was reverse-transcribed using stem-loop Multiplex primer pools (Applied Biosystems, Dublin, Ireland). We used primers for the mmu-miR-134 (Applied Biosystems miRNA assay ID 00186), and real-time quantitative PCR was carried out on a 7900HT Fast Realtime System (Applied Biosystems) using TaqMan miRNA assays (Applied Biosystems). U19 (Applied Biosystems miRNA assay ID 001003) was used for normalization. A relative fold change in expression of the target gene transcript was determined using the comparative cycle threshold method ($2-^{\Delta\Delta}$CT). For analysis of protein-coding transcripts, cDNA was produced from 500 ng total RNA by reverse transcription using Superscript III Reverse Transcriptase enzyme (Invitrogen). Quantitative PCR was performed using a LightCycler 1.5 (Roche Diagnostics) and QuantiTech SYBR Green PCR kit (Qiagen) as per the manufacturer's instructions, and 25 μM of primer mix was used. Specific primers for each gene assayed were purchased from Sigma, and sequences used were DCX forward: tttcccagggtcatgttgca, reverse: tgctcaacgtctcaatgcct; Human_Creb1 forward: ttgcaaaacacaggcccaag, reverse: atttgatgt caggcccgtgt; Human LimK-1 forward: agtgagctttgccaaggaca, reverse: ttcttgcggtctggcttctt; Human Serpine1 forward: tccccagaaa cagtgtgcat, reverse: aagcactcaagggcaaggat; β-actin forward: agcgag catcccccaaagtt, reverse: gggcacgaaggctcatcatt was used for normalization and relative mRNA transcript levels assessed using the standard $^{\Delta\Delta}$CT method.

Ube3a isoform expression analysis

Expression levels of specific *Ube3a/UBE3A* splicing variants were measured, specifically the putative miRNA sponge variant (isoform with an early 3′ UTR after exon 7) and the protein coding variants (protein coding isoforms containing the remaining exons downstream of exon 7). This was achieved by retrieving publicly available RNA-seq data and by performing qRT-PCR on mouse and human iPSC samples. RNA-seq count tables were retrieved from the Gene Expression Omnibus. Mouse hippocampus data was retrieved from entries GSE187651 (2x P36 male mice) and GSE187304 (2x P36 female mice). Both mouse datasets were generated by the ENCODE Project Consortium.[64] Human brain RNA-seq data (lateral motor cortex) data were retrieved from entry GSE138053, specifically samples GSM4097470 and GSM4097471.[65] Mouse expression values were normalized by calculating $\log_2$ of the counts per million (CPM) + 1 of each variant.

For qRT-PCR, hippocampi of P24 WT and *Ube3a*$^{m-/p+}$ mice, along with control (n = 9) and AS (n = 9) human iPSCs were used. RNA (1 μg) was treated with DNAse I (Invitrogen) and reverse-transcribed using Superscript III (Invitrogen) along with random hexamer primers. Real-time quantitative PCR was performed on a QuantStudio 12 system (Applied Biosystems), along with TaqMan Fast 2X Master Mix (Applied Biosystems) and PrimeTime Probe Assays (Integrated DNA Technologies). Primers were designed to detect either the putative miRNA sponge variant or the protein coding variants. The sequences used were the following: mouse putative miRNA sponge: forward: tgtggaagccggaatctaga, reverse: acaggacacaaaagcacaca, probe: atgacggtggctatacgagg; mouse protein coding: forward: tgtgattagggagttctggga, reverse: gtaacctttctgtgtctgggc, probe: tgcagttta caacaggcaca; human putative miRNA sponge: forward: acaggaag gaatttgtcaatctt, reverse: gaagcaaaatcacacacccct, probe: tcaaggcttttcg gagaggt; human protein coding: forward: tctgattagggagttctggga, reverse: ctttctgtgtctgggccatt, probe – tcttgcagtttacaacgggc.

Statistical analysis

Statistical analysis was performed using GraphPad Prism (version 8). Data were tested for normality using Shapiro-Wilk test. Normally distributed datasets were presented as mean ± SEM and analyzed using the two-tailed Student's t test or one-way ANOVA with Bonferroni post hoc testing, as appropriate. Non-normally distributed datasets were presented as median ± IQR and analyzed using the two-tailed Mann-Whitney U test or Kruskal-Wallis test with Dunn's post hoc testing, as appropriate. The individual tests used for each comparison are specified in the text. Alpha was set to 0.05 for all tests used. Analysis of the Racine scale was performed using Stata Release 16.1. Mortality was compared between treatments using incidence rate ratios and a Poisson model, with number of trials as the exposure variable. Seizure severity was modeled using ordinal logistic regression with robust variance estimation used to adjust for clustering of data with mice. An interaction term was used to test for a change in effectiveness of treatment as a function of trial number. For miRNA and transcripts in iPSC studies, two-group comparisons were made using unpaired two-tailed Student's t test.

SUPPLEMENTAL INFORMATION

Supplemental information can be found online at https://doi.org/10.1016/j.omtn.2022.04.009.

ACKNOWLEDGMENTS

We thank Lisa-Ann Byrne for support with ethical and licensing aspects of the research, Tobias Engel for support with iPSCs, and Tammy Strickland for help with illustrations. This publication has emanated from research supported in part by a research grant from Science Foundation Ireland (SFI) under grant 16/RC/3948 and co-funded under the European Regional Development Fund and by FutureNeuro industry partners (F. Hoffman-La Roche Ltd.). The present study was also supported by funding from the RCSI's STAR PhD program (to A.C.). C.R.R. acknowledges funding from the Irish Research Council (GOIPD/2014/566), CURE Epilepsy Cameron Boyce Foundation Taking Flight Award, and Science Foundation Ireland (17/TIDA/5002). G.M. is supported by a Marie Skłodowska-Curie Actions Individual Fellowship ("EpimiRTherapy," H2020-MSCA-IF-2018 840262) and J.C.K. by SFI award 17/CDA/4708.

AUTHOR CONTRIBUTIONS

A.C., G.M., A.S., E.L., J.C.K., N.T.N., and C.R.R. generated and/or analyzed the experimental data. A.C. and J.A. managed the mouse colony and performed genotypic characterization. L.K. designed the antimiR. A.C. and R.M.C. performed statistical analysis. A.C., C.R.R., M.T.M., J.W., M.A.J., and D.C.H. designed the project. D.C.H. and A.C. wrote the paper.

DECLARATION OF INTERESTS

The Royal College of Surgeons in Ireland (D.C.H.) holds a patent for the inhibition of microRNA-134 for the treatment of seizure-related disorders and other neurologic injuries (US 9,803,200 B2). J.W., M.A.J., and M.T.M. are employees of F Hoffman-La Roche Ltd. All other authors have no competing interests.

REFERENCES

1. Buiting, K., Williams, C., and Horsthemke, B. (2016). Angelman syndrome - insights into a rare neurogenetic disorder. Nat. Rev. Neurol. *12*, 584–593. https://doi.org/10.1038/nrneurol.2016.133.
2. Pelc, K., Boyd, S.G., Cheron, G., and Dan, B. (2008). Epilepsy in Angelman syndrome. Seizure *17*, 211–217. https://doi.org/10.1016/j.seizure.2007.08.004.
3. Margolis, S.S., Sell, G.L., Zbinden, M.A., and Bird, L.M. (2015). Angelman syndrome. Neurotherapeutics *12*, 641–650. https://doi.org/10.1007/s13311-015-0361-y.
4. Huang, H.S., Allen, J.A., Mabb, A.M., King, I.F., Miriyala, J., Taylor-Blake, B., Sciaky, N., Dutton, J.W., Jr., Lee, H.M., Chen, X., et al. (2011). Topoisomerase inhibitors unsilence the dormant allele of Ube3a in neurons. Nature *481*, 185–189. https://doi.org/10.1038/nature10726.
5. Meng, L., Ward, A.J., Chun, S., Bennett, C.F., Beaudet, A.L., and Rigo, F. (2015). Towards a therapy for Angelman syndrome by targeting a long non-coding RNA. Nature *518*, 409–412. https://doi.org/10.1038/nature13975.
6. Wolter, J.M., Mao, H., Fragola, G., Simon, J.M., Krantz, J.L., Bazick, H.O., Oztemiz, B., Stein, J.L., and Zylka, M.J. (2020). Cas9 gene therapy for Angelman syndrome traps Ube3a-ATS long non-coding RNA. Nature *587*, 281–284. https://doi.org/10.1038/s41586-020-2835-2.
7. Schmid, R.S., Deng, X., Panikker, P., Msackyi, M., Breton, C., and Wilson, J.M. (2021). CRISPR/Cas9 directed to the Ube3a antisense transcript improves Angelman syndrome phenotype in mice. J. Clin. Invest. *131*, e142574. https://doi.org/10.1172/jci142574.
8. Silva-Santos, S., van Woerden, G.M., Bruinsma, C.F., Mientjes, E., Jolfaei, M.A., Distel, B., Kushner, S.A., and Elgersma, Y. (2015). Ube3a reinstatement identifies distinct developmental windows in a murine Angelman syndrome model. J. Clin. Invest. *125*, 2069–2076. https://doi.org/10.1172/jci80554.
9. Gu, B., Carstens, K.E., Judson, M.C., Dalton, K.A., Rougie, M., Clark, E.P., Dudek, S.M., and Philpot, B.D. (2019). Ube3a reinstatement mitigates epileptogenesis in Angelman syndrome model mice. J. Clin. Invest. *129*, 163–168. https://doi.org/10.1172/jci120816.
10. Cruz, E., Descalzi, G., Steinmetz, A., Scharfman, H.E., Katzman, A., and Alberini, C.M. (2021). CIM6P/IGF-2 receptor ligands reverse deficits in Angelman syndrome model mice. Autism Res. *14*, 29–45. https://doi.org/10.1002/aur.2418.
11. Valluy, J., Bicker, S., Aksoy-Aksel, A., Lackinger, M., Sumer, S., Fiore, R., Wust, T., Seffer, D., Metge, F., Dieterich, C., et al. (2015). A coding-independent function of an alternative Ube3a transcript during neuronal development. Nat. Neurosci. *18*, 666–673. https://doi.org/10.1038/nn.3996.
12. Gebert, L.F.R., and MacRae, I.J. (2019). Regulation of microRNA function in animals. Nat. Rev. Mol. Cell Biol. *20*, 21–37. https://doi.org/10.1038/s41580-018-0045-7.
13. Chandradoss, S.D., Schirle, N.T., Szczepaniak, M., MacRae, I.J., and Joo, C. (2015). A dynamic search process underlies microRNA targeting. Cell *162*, 96–107. https://doi.org/10.1016/j.cell.2015.06.032.
14. Jimenez-Mateos, E.M., Engel, T., Merino-Serrais, P., McKiernan, R.C., Tanaka, K., Mouri, G., Sano, T., O'Tuathaigh, C., Waddington, J.L., Prenter, S., et al. (2012). Silencing microRNA-134 produces neuroprotective and prolonged seizure-suppressive effects. Nat. Med. *18*, 1087–1094. https://doi.org/10.1038/nm.2834.
15. Peng, J., Omran, A., Ashhab, M.U., Kong, H., Gan, N., He, F., and Yin, F. (2013). Expression patterns of miR-124, miR-134, miR-132, and miR-21 in an immature rat model and children with mesial temporal lobe epilepsy. J. Mol. Neurosci. *50*, 291–297. https://doi.org/10.1007/s12031-013-9953-3.
16. Jimenez-Mateos, E.M., Engel, T., Merino-Serrais, P., Fernaud-Espinosa, I., Rodriguez-Alvarez, N., Reynolds, J., Reschke, C.R., Conroy, R.M., McKiernan, R.C., deFelipe, J., and Henshall, D.C. (2015). Antagomirs targeting microRNA-134 increase hippocampal pyramidal neuron spine volume in vivo and protect against pilocarpine-induced status epilepticus. Brain Struc. Funct. *220*, 2387–2399. https://doi.org/10.1007/s00429-014-0798-5.
17. Venø, M.T., Reschke, C.R., Morris, G., Connolly, N.M.C., Su, J., Yan, Y., Engel, T., Jimenez-Mateos, E.M., Harder, L.M., Pultz, D., et al. (2020). A systems approach delivers a functional microRNA catalog and expanded targets for seizure suppression in temporal lobe epilepsy. Proc. Natl. Acad. Sci. U S A. *117*, 15977–15988. https://doi.org/10.1073/pnas.1919313117.
18. Sheinerman, K.S., Tsivinsky, V.G., Crawford, F., Mullan, M.J., Abdullah, L., and Umansky, S.R. (2012). Plasma microRNA biomarkers for detection of mild cognitive impairment. Aging *4*, 590–605. https://doi.org/10.18632/aging.100486.
19. Schratt, G.M., Tuebing, F., Nigh, E.A., Kane, C.G., Sabatini, M.E., Kiebler, M., and Greenberg, M.E. (2006). A brain-specific microRNA regulates dendritic spine development. Nature *439*, 283–289. https://doi.org/10.1038/nature04367.
20. Gao, J., Wang, W.Y., Mao, Y.W., Graff, J., Guan, J.S., Pan, L., Mak, G., Kim, D., Su, S.C., and Tsai, L.H. (2010). A novel pathway regulates memory and plasticity via SIRT1 and miR-134. Nature *466*, 1105–1109. https://doi.org/10.1038/nature09271.
21. Gaughwin, P., Ciesla, M., Yang, H., Lim, B., and Brundin, P. (2011). Stage-specific modulation of cortical neuronal development by Mmu-miR-134. Cereb. Cortex *21*, 1857–1869. https://doi.org/10.1093/cercor/bhq262.
22. Wang, X.M., Jia, R.H., Wei, D., Cui, W.Y., and Jiang, W. (2014). MiR-134 blockade prevents status epilepticus like-activity and is neuroprotective in cultured hippocampal neurons. Neurosci. Lett. *572*, 20–25. https://doi.org/10.1016/j.neulet.2014.04.049.
23. Reschke, C.R., Silva, L.F.A., Norwood, B.A., Senthilkumar, K., Morris, G., Sanz-Rodriguez, A., Conroy, R.M., Costard, L., Neubert, V., Bauer, S., et al. (2017). Potent anti-seizure effects of locked nucleic acid antagomirs targeting miR-134 in multiple mouse and rat models of epilepsy. Mol. Ther. Nucleic Acid. *6*, 45–56. https://doi.org/10.1016/j.omtn.2016.11.002.

24. Gao, X., Guo, M., Meng, D., Sun, F., Guan, L., Cui, Y., Zhao, Y., Wang, X., Gu, X., Sun, J., and Qi, S. (2019). Silencing microRNA-134 alleviates hippocampal damage and occurrence of spontaneous seizures after intraventricular kainic acid-induced status epilepticus in rats. Front. Cell Neurosci. *13*, 145. https://doi.org/10.3389/fncel.2019.00145.

25. Jiang, Y.H., Armstrong, D., Albrecht, U., Atkins, C.M., Noebels, J.L., Eichele, G., Sweatt, J.D., and Beaudet, A.L. (1998). Mutation of the Angelman ubiquitin ligase in mice causes increased cytoplasmic p53 and deficits of contextual learning and long-term potentiation. Neuron *21*, 799–811. https://doi.org/10.1016/s0896-6273(00)80596-6.

26. Sonzogni, M., Wallaard, I., Santos, S.S., Kingma, J., du Mee, D., van Woerden, G.M., and Elgersma, Y. (2018). A behavioral test battery for mouse models of Angelman syndrome: a powerful tool for testing drugs and novel Ube3a mutants. Mol. Autism *9*, 47. https://doi.org/10.1186/s13229-018-0231-7.

27. Sidorov, M.S., Deck, G.M., Dolatshahi, M., Thibert, R.L., Bird, L.M., Chu, C.J., and Philpot, B.D. (2017). Delta rhythmicity is a reliable EEG biomarker in Angelman syndrome: a parallel mouse and human analysis. J. Neurodev. Disord. *9*, 17. https://doi.org/10.1186/s11689-017-9195-8.

28. Reschke, C.R., Silva, L.F.A., Vangoor, V.R., Rosso, M., David, B., Cavanagh, B.L., Connolly, N.M.C., Brennan, G.P., Sanz-Rodriguez, A., Mooney, C., et al. (2021). Systemic delivery of antagomirs during blood-brain barrier disruption is disease-modifying in experimental epilepsy. Mol. Ther. *29*, 2041–2052. https://doi.org/10.1016/j.ymthe.2021.02.021.

29. Fiore, R., Khudayberdiev, S., Christensen, M., Siegel, G., Flavell, S.W., Kim, T.K., Greenberg, M.E., and Schratt, G. (2009). Mef2-mediated transcription of the miR379-410 cluster regulates activity-dependent dendritogenesis by fine-tuning Pumilio2 protein levels. EMBO J. *28*, 697–710. https://doi.org/10.1038/emboj.2009.10.

30. Wu, Y.P., Siao, C.J., Lu, W., Sung, T.C., Frohman, M.A., Milev, P., Bugge, T.H., Degen, J.L., Levine, J.M., Margolis, R.U., and Tsirka, S.E. (2000). The tissue plasminogen activator (tPA)/plasmin extracellular proteolytic system regulates seizure-induced hippocampal mossy fiber outgrowth through a proteoglycan substrate. J. Cell Biol. *148*, 1295–1304. https://doi.org/10.1083/jcb.148.6.1295.

31. Flores, O., Kennedy, E.M., Skalsky, R.L., and Cullen, B.R. (2014). Differential RISC association of endogenous human microRNAs predicts their inhibitory potential. Nucleic Acid. Res. *42*, 4629–4639. https://doi.org/10.1093/nar/gkt1393.

32. Campbell, A., Morris, G., Heller, J.P., Langa, E., Brindley, E., Worm, J., Jensen, M.A., Miller, M.T., Henshall, D.C., and Reschke, C.R. (2021). Antagomir-mediated suppression of microRNA-134 reduces kainic acid-induced seizures in immature mice. Sci. Rep. *11*, 340. https://doi.org/10.1038/s41598-020-79350-7.

33. Fuller, J.L., and Sjursen, F.H., Jr. (1967). Audiogenic seizures in eleven mouse strains. J. Hered. *58*, 135–140. https://doi.org/10.1093/oxfordjournals.jhered.a107565.

34. Mandel-Brehm, C., Salogiannis, J., Dhamne, S.C., Rotenberg, A., and Greenberg, M.E. (2015). Seizure-like activity in a juvenile Angelman syndrome mouse model is attenuated by reducing Arc expression. Proc. Natl. Acad. Sci. U S A. *112*, 5129–5134. https://doi.org/10.1073/pnas.1504809112.

35. Pandya, N.J., Wang, C., Costa, V., Lopatta, P., Meier, S., Zampeta, F.I., Punt, A.M., Mientjes, E., Grossen, P., Distler, T., et al. (2021). Secreted retrovirus-like GAG-domain–containing protein PEG10 is regulated by UBE3A and is involved in Angelman syndrome pathophysiology. Cell Rep. Med. *2*, 100360. https://doi.org/10.1016/j.xcrm.2021.100360.

36. Whipple, A.J., Breton-Provencher, V., Jacobs, H.N., Chitta, U.K., Sur, M., and Sharp, P.A. (2020). Imprinted maternally expressed microRNAs antagonize paternally driven gene programs in neurons. Mol. Cell *78*, 85–95.e8. https://doi.org/10.1016/j.molcel.2020.01.020.

37. Bicker, S., Lackinger, M., Weiß, K., and Schratt, G. (2014). MicroRNA-132, -134, and -138: a microRNA troika rules in neuronal dendrites. Cell Mol. Life Sci. *71*, 3987–4005. https://doi.org/10.1007/s00018-014-1671-7.

38. Morris, G., Reschke, C.R., and Henshall, D.C. (2019). Targeting microRNA-134 for seizure control and disease modification in epilepsy. EBioMedicine *45*, 646–654. https://doi.org/10.1016/j.ebiom.2019.07.008.

39. Dindot, S.V., Antalffy, B.A., Bhattacharjee, M.B., and Beaudet, A.L. (2008). The Angelman syndrome ubiquitin ligase localizes to the synapse and nucleus, and maternal deficiency results in abnormal dendritic spine morphology. Hum. Mol. Genet. *17*, 111–118. https://doi.org/10.1093/hmg/ddm288.

40. Streng, M.L., and Krook-Magnuson, E. (2021). The cerebellum and epilepsy. Epilepsy Behav. *121*, 106909. https://doi.org/10.1016/j.yebeh.2020.106909.

41. Janssen, H.L., Reesink, H.W., Lawitz, E.J., Zeuzem, S., Rodriguez-Torres, M., Patel, K., van der Meer, A.J., Patick, A.K., Chen, A., Zhou, Y., et al. (2013). Treatment of HCV infection by targeting microRNA. N. Engl. J. Med. *368*, 1685–1694. https://doi.org/10.1056/nejmoa1209026.

42. Morris, G., O'Brien, D., and Henshall, D.C. (2021). Opportunities and challenges for microRNA-targeting therapeutics for epilepsy. Trends Pharmacol. Sci. *42*, 605–616. https://doi.org/10.1016/j.tips.2021.04.007.

43. Markati, T., Duis, J., and Servais, L. (2021). Therapies in preclinical and clinical development for Angelman syndrome. Expert Opin. Invest. Drugs *30*, 709–720. https://doi.org/10.1080/13543784.2021.1939674.

44. Deverman, B.E., Ravina, B.M., Bankiewicz, K.S., Paul, S.M., and Sah, D.W.Y. (2018). Gene therapy for neurological disorders: progress and prospects. Nat. Rev. Drug Discov. *17*, 641–659. https://doi.org/10.1038/nrd.2018.110.

45. Prut, L., and Belzung, C. (2003). The open field as a paradigm to measure the effects of drugs on anxiety-like behaviors: a review. Eur. J. Pharmacol. *463*, 3–33. https://doi.org/10.1016/s0014-2999(03)01272-x.

46. Swahari, V., Nakamura, A., Hollville, E., Stroud, H., Simon, J.M., Ptacek, T.S., Beck, M.V., Flowers, C., Guo, J., Plestant, C., et al. (2021). MicroRNA-29 is an essential regulator of brain maturation through regulation of CH methylation. Cell Rep. *35*, 108946. https://doi.org/10.1016/j.celrep.2021.108946.

47. Corbo, J.C., Deuel, T.A., Long, J.M., LaPorte, P., Tsai, E., Wynshaw-Boris, A., and Walsh, C.A. (2002). Doublecortin is required in mice for lamination of the hippocampus but not the neocortex. J. Neurosci. *22*, 7548–7557. https://doi.org/10.1523/jneurosci.22-17-07548.2002.

48. Valverde, O., Mantamadiotis, T., Torrecilla, M., Ugedo, L., Pineda, J., Bleckmann, S., Gass, P., Kretz, O., Mitchell, J.M., Schutz, G., and Maldonado, R. (2004). Modulation of anxiety-like behavior and morphine dependence in CREB-deficient mice. Neuropsychopharmacology *29*, 1122–1133. https://doi.org/10.1038/sj.npp.1300416.

49. Pierce, K., and Courchesne, E. (2001). Evidence for a cerebellar role in reduced exploration and stereotyped behavior in autism. Biol. Psych. *49*, 655–664. https://doi.org/10.1016/s0006-3223(00)01008-8.

50. Bauer, D.J., Kerr, A.L., and Swain, R.A. (2011). Cerebellar dentate nuclei lesions reduce motivation in appetitive operant conditioning and open field exploration. Neurobiol. Learn. Mem. *95*, 166–175. https://doi.org/10.1016/j.nlm.2010.12.009.

51. Avagliano Trezza, R., Sonzogni, M., Bossuyt, S.N.V., Zampeta, F.I., Punt, A.M., van den Berg, M., Rotaru, D.C., Koene, L.M.C., Munshi, S.T., Stedehouder, J., et al. (2019). Loss of nuclear UBE3A causes electrophysiological and behavioral deficits in mice and is associated with Angelman syndrome. Nat. Neurosci. *22*, 1235–1247. https://doi.org/10.1038/s41593-019-0425-0.

52. Rago, L., Beattie, R., Taylor, V., and Winter, J. (2014). miR379-410 cluster miRNAs regulate neurogenesis and neuronal migration by fine-tuning N-cadherin. EMBO J. *33*, 906–920. https://doi.org/10.1002/embj.201386591.

53. Marty, V., Labialle, S., Bortolin-Cavaille, M.L., Ferreira De Medeiros, G., Moisan, M.P., Florian, C., and Cavaille, J. (2016). Deletion of the miR-379/miR-410 gene cluster at the imprinted Dlk1-Dio3 locus enhances anxiety-related behaviour. Hum. Mol. Genet. *25*, 728–739. https://doi.org/10.1093/hmg/ddv510.

54. Lackinger, M., Sungur, A.O., Daswani, R., Soutschek, M., Bicker, S., Stemmler, L., Wust, T., Fiore, R., Dieterich, C., Schwarting, R.K., et al. (2019). A placental mammal-specific microRNA cluster acts as a natural brake for sociability in mice. EMBO Rep. *20*, e46429. https://doi.org/10.15252/embr.201846429.

55. Bekenstein, U., Mishra, N., Milikovsky, D.Z., Hanin, G., Zelig, D., Sheintuch, L., Berson, A., Greenberg, D.S., Friedman, A., and Soreq, H. (2017). Dynamic changes in murine forebrain miR-211 expression associate with cholinergic imbalances and epileptiform activity. Proc. Natl. Acad. Sci. U S A. *114*, E4996–E5005. https://doi.org/10.1073/pnas.1701201114.

56. Tucci, V., Isles, A.R., Kelsey, G., Ferguson-Smith, A.C., Erice Imprinting, G., Bartolomei, M.S., Benvenisty, N., Bourc'his, D., Charalambous, M., Dulac, C., et al. (2019). Genomic imprinting and physiological processes in mammals. Cell *176*, 952–965. https://doi.org/10.1016/j.cell.2019.01.043.

57. Pober, B.R. (2010). Williams-Beuren syndrome. N. Engl. J. Med. *362*, 239–252. https://doi.org/10.1056/nejmra0903074.

58. Fujitani, M., Zhang, S., Fujiki, R., Fujihara, Y., and Yamashita, T. (2017). A chromosome 16p13.11 microduplication causes hyperactivity through dysregulation of miR-484/protocadherin-19 signaling. Mol. Psych. *22*, 364–374. https://doi.org/10.1038/mp.2016.106.

59. Carballosa-Gonzalez, M.M., Munoz, L.J., Lopez-Alburquerque, T., Pardal-Fernandez, J.M., Nava, E., de Cabo, C., Sancho, C., and Lopez, D.E. (2013). EEG characterization of audiogenic seizures in the hamster strain GASH:Sal. Epilepsy Res. *106*, 318–325. https://doi.org/10.1016/j.eplepsyres.2013.07.001.

60. Wong, N., and Wang, X. (2015). miRDB: an online resource for microRNA target prediction and functional annotations. Nucleic Acids Res. *43*, D146–D152. https://doi.org/10.1093/nar/gku1104.

61. Hill, M.R., and Greenfield, S.A. (2011). The membrane chamber: a new type of in vitro recording chamber. J. Neurosci. Meth. *195*, 15–23. https://doi.org/10.1016/j.jneumeth.2010.10.024.

62. Morris, G., Jiruska, P., Jefferys, J.G.R., and Powell, A.D. (2016). A new approach of modified submerged patch clamp recording reveals interneuronal dynamics during epileptiform oscillations. Front. Neurosci. *10*, 519. https://doi.org/10.3389/fnins.2016.00519.

63. Schindelin, J., Arganda-Carreras, I., Frise, E., Kaynig, V., Longair, M., Pietzsch, T., Preibisch, S., Rueden, C., Saalfeld, S., Schmid, B., et al. (2012). Fiji: an open-source platform for biological-image analysis. Nat. Meth. *9*, 676–682. https://doi.org/10.1038/nmeth.2019.

64. Consortium, E.P. (2012). An integrated encyclopedia of DNA elements in the human genome. Nature *489*, 57–74. https://doi.org/10.1038/nature11247.

65. Lim, W.F., Forouhan, M., Roberts, T.C., Dabney, J., Ellerington, R., Speciale, A.A., Manzano, R., Lieto, M., Sangha, G., Banerjee, S., et al. (2021). Gene therapy with AR isoform 2 rescues spinal and bulbar muscular atrophy phenotype by modulating AR transcriptional activity. Sci. Adv. *7*, eabi6896. https://doi.org/10.1126/sciadv.abi6896.

OMTN, Volume ***28***

Supplemental information

AntimiR targeting of microRNA-134 reduces seizures in a mouse model of Angelman syndrome

Aoife Campbell, Gareth Morris, Albert Sanfeliu, Joana Augusto, Elena Langa, Jaideep C. Kesavan, Ngoc T. Nguyen, Ronan M. Conroy, Jesper Worm, Lukasz Kielpinski, Mads Aaboe Jensen, Meghan T. Miller, Thomas Kremer, Cristina R. Reschke, and David C. Henshall

Table S1: Upregulated DEGs of interest between Ant-134 and Scr-treated Ube3a mice. DEGs of interest include predicted miR-134 targets, genes previously identified as differentially expressed after Ant-134 treatment [*Reschke et al, Mol Ther 2021*][28] and genes associated with epilepsy.

Gene Symbol	log2 Fold Change	p-value	Previously identified Ant-134 target	miR-134 target	Epilepsy associated
Scn3b	0.14	0.0004	NO	NO	YES
Ar	0.23	0.0011	NO	NO	YES
Chrm1	0.11	0.0021	NO	NO	YES
Drd5	0.30	0.0036	NO	YES	NO
Ldb3	0.61	0.0042	NO	YES	NO
Gstm1	0.19	0.0043	NO	NO	YES
Arpc2	0.13	0.0053	NO	NO	YES
Gtf2h1	0.16	0.0056	NO	YES	NO
Zmynd8	0.13	0.0082	NO	NO	YES
Chn1	0.12	0.0087	NO	NO	YES
Fer	0.23	0.0109	NO	YES	NO
Calm2	0.10	0.0124	NO	NO	YES
Lman2l	0.16	0.0139	NO	NO	YES
Ccdc6	0.13	0.0164	NO	NO	YES
Grem1	0.37	0.0178	NO	YES	YES
Crb1	0.79	0.0188	NO	NO	YES
Dapk1	0.12	0.0193	NO	NO	YES
Pcp4	0.29	0.0201	NO	NO	YES
Kctd21	0.20	0.0204	NO	YES	NO
Cox10	0.14	0.0212	NO	NO	YES
Tnfsf14	0.96	0.0236	NO	NO	YES
Snap25	0.12	0.0260	NO	NO	YES
Eno2	0.10	0.0269	NO	NO	YES
Suclg1	0.11	0.0280	NO	NO	YES
Tbl1xr1	0.12	0.0290	NO	NO	YES
Ppia	0.21	0.0296	NO	NO	YES
Dlg3	0.09	0.0296	NO	NO	YES
Katnal1	0.12	0.0299	NO	YES	NO
Gatad2b	0.09	0.0304	NO	NO	YES
Hspd1	0.10	0.0305	NO	NO	YES
Nptn	0.07	0.0311	NO	NO	YES
Acadsb	0.10	0.0339	NO	NO	YES
Bhlhe22	0.17	0.0355	NO	NO	YES
Ppp3ca	0.10	0.0364	NO	NO	YES
Ndufaf1	0.18	0.0371	NO	NO	YES

Smarca2	0.08	0.0377	NO	NO	YES
Ndufa11	0.14	0.0378	NO	NO	YES
Ptgs2	0.23	0.0382	NO	NO	YES
Gabrg2	0.09	0.0390	NO	NO	YES
Npy1r	0.15	0.0402	NO	YES	NO
Snca	0.10	0.0409	NO	NO	YES
Glo1	0.09	0.0433	NO	NO	YES
Hsp90ab1	0.07	0.0441	NO	NO	YES
Ywhae	0.06	0.0445	NO	NO	YES
Gpt2	0.13	0.0470	NO	NO	YES
Eef1a2	0.08	0.0473	NO	NO	YES
Lat	0.59	0.0474	NO	NO	YES
Ier3	0.34	0.0476	YES	NO	NO
Mfsd8	0.17	0.0482	NO	NO	YES
Sgce	0.15	0.0490	NO	NO	YES
Tpo	0.55	0.0499	NO	NO	YES
Slc1a1	0.08	0.0499	NO	NO	YES

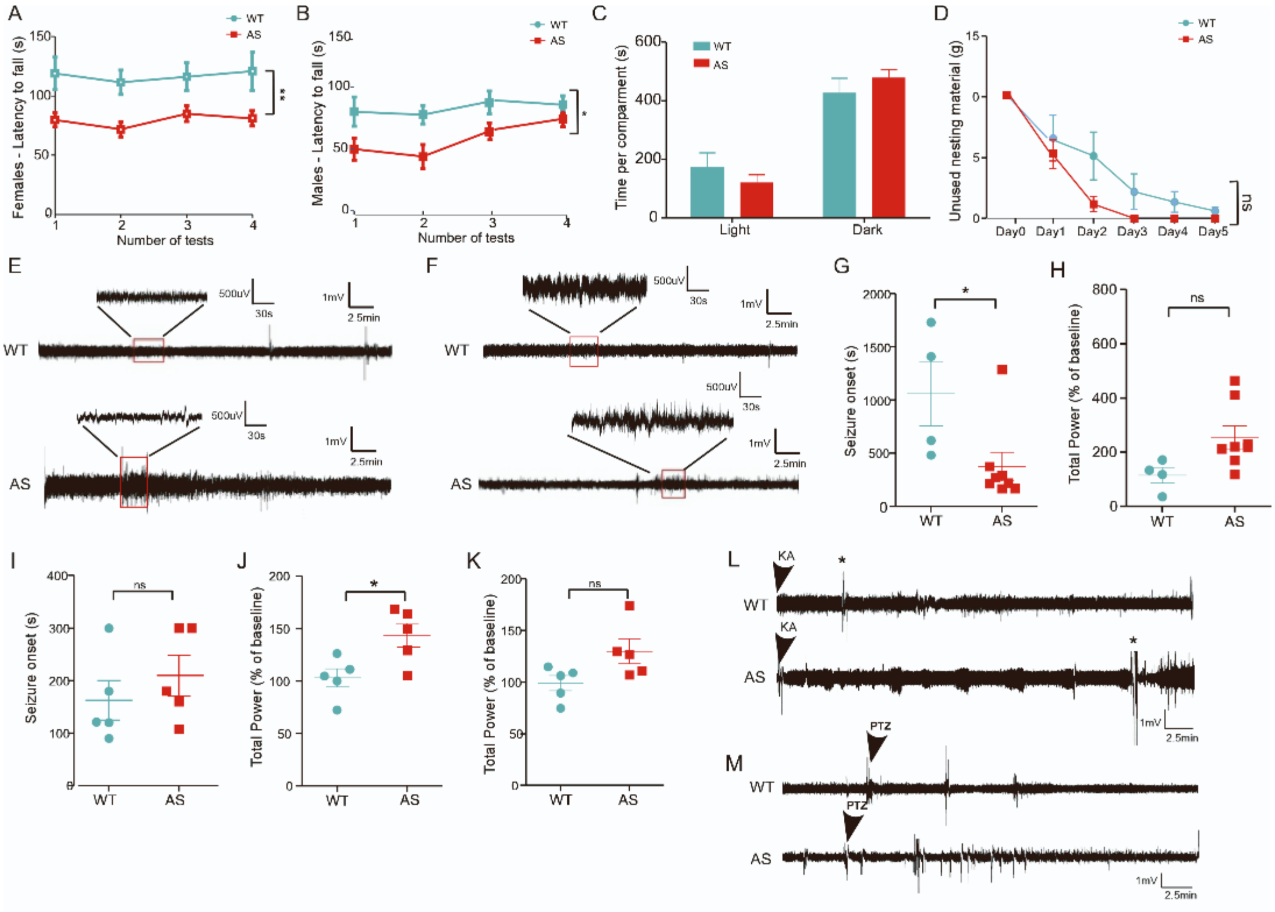

Figure S1: Additional features of F1 generation AS mice. Analysis of behavioural and seizure sensitivity phenotype in P42 F1 *Ube3a*$^{m-/p+}$ mice. **(A-B)** Graphs show the time spent on the rotarod during four test phases in **(A)** female mice, WT n=7, AS n=10, p=0.004 and **(B)** in male mice, WT n=7, AS n=10, p=0.015. **(C)** Graphs shows the length of time (s) spent in dark and light compartment during the 10 minute trial, WT n=8, AS n=14, p=0.16. **(D)** Graph shows the amount of unused nesting material from day 0 to day 5, WT n=3, AS n=4, p=0.16. **(E-F)** Representative EEG traces of 1 hour recording from **(E)** P21 and **(F)** adult WT and AS mice, Scale bar 1 mV, 2.5 min for full recording, 500 uV, 30 s for zoom. **(G)** Time (s) to the first electrographic seizure following injection of a low dose of KA (20 mg/kg, i.p.), WT *n*=4, AS *n*=8, p=0.0162. **(H)** Total power increase (%) from its own baseline when recording ictal activity following injection of 20 mg/kg KA, WT *n*=4, AS *n*=8, p=0.053. **(I)** Time (s) to the first electrographic seizure following low dose PTZ (40 mg/kg, i.p.), WT n=5, AS n=5, p=0.457. **(J)** Total power (% of increase from each mouse own baseline) from the first 10 minutes after injection 40 mg/kg PTZ, WT n=5, AS n=5, p=0.024**. (K)** Total power (% baseline) from entire 30 minute recording after injection 40 mg/kg PTZ, WT n=5, AS n=5, p=0.059**. (L-M)** Representative EEG traces of WT and AS mice following **(L)** 20 mg/kg KA or **(M)** 40 mg/kg PTZ administration (black arrows), Scale bar 1 mV, 2.5 min. The Shapiro-Wilk test was used to test for normality, and *t*-test or a Mann-Whitney test was performed for statistical analysis. Data are expressed as mean ± SEM.

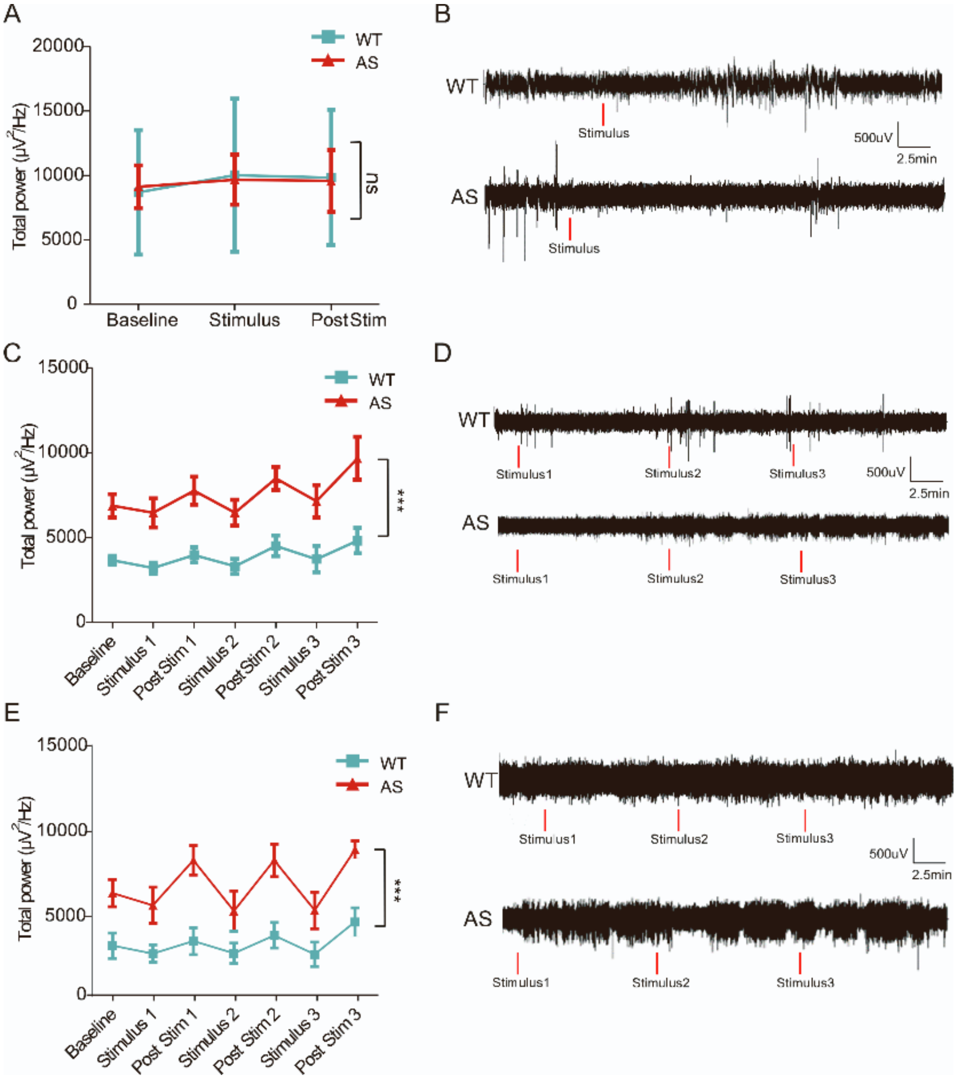

Figure S2: Inability to elicit audiogenic seizures in F1 generation AS mice. F1 WT and AS mice were used in attempt to generate an audiogenic seizure model at three different ages. **(A)** No changes in the EEG total power ($\mu V^2 Hz$) at baseline, during stimulus and post-stimulus were observed in P21 mice, WT n=3, AS n=3, p=0.99. **(B)** Representative EEG traces of right hemisphere recordings from WT and AS P21 mice. **(C-F)** Although significant changes in the total EEG power ($\mu V^2 Hz$) were observed at baseline, during and after stimuli in **(C)** P28 (WT *n*=4, AS *n*=8, ***p<0.001) and **(E)** in P42 mice (WT n=3, AS n=3, ***p<0.001), no audiogenic-induced clinical (behavioural) seizures were observed. Representative EEG traces of the right brain hemisphere recordings in WT and AS **(D)** P28 and **(F)** P42 mice depict pre- and post- audiogenic stimulus periods. Data are represented as mean ± SEM. A Shapiro-Wilk test was used to check normality of data and two-way ANOVA was used for statistical analysis.

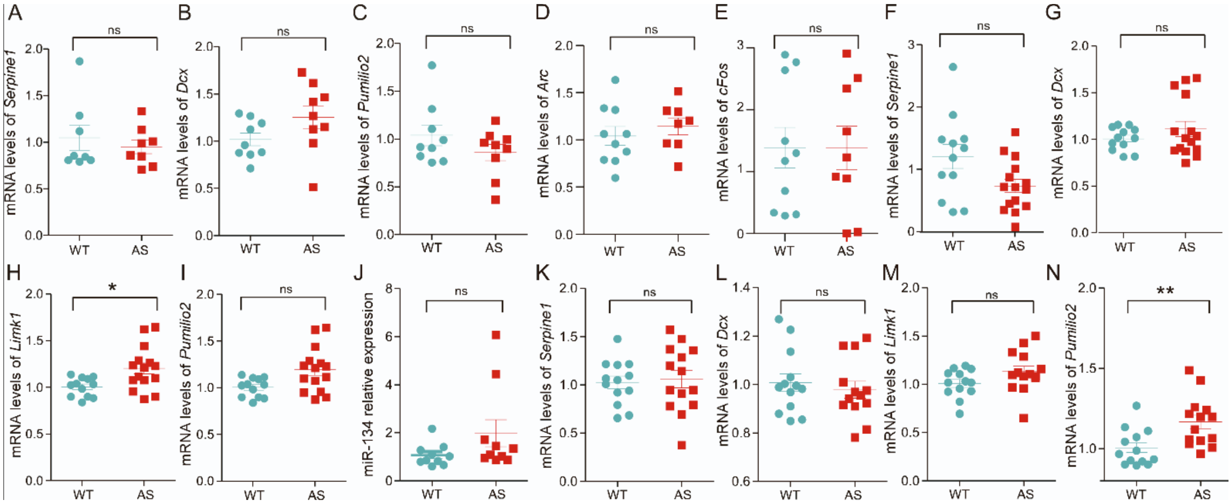

Figure S3: **Additional gene expression analyses in AS mice.** Transcript analysis of levels of miR-134 and its targets from the **(A-E)** cerebellum, **(F-I)** hippocampus and **(J-N)** cortex of naïve P21 F1 generation mice. **(A)** mRNA levels of *Serpine1* in the cerebellum of P21 mice, WT n=8, AS n=8, p=0.54. **(B)** mRNA levels of *Dcx* in the cerebellum of P21 mice, WT n=9, AS n=9, p=0.11. **(C)** mRNA levels of *Pumilio2* in the cerebellum of P21 mice, WT n=9, AS n=9, p=0.21. **(D)** mRNA levels of *Arc* in the cerebellum of P21 mice, WT n=10, AS n=9, p=0.470 **(E)** mRNA levels of *cFos* in the cerebellum of P21 mice, WT n=10, AS, n=9, p=0.99 **(F)** mRNA levels of *Serpine1* in the hippocampus of P21 mice, WT n=12, AS n=15, p=0.037. **(G)** mRNA levels of *Dcx* in the hippocampus of P21 mice, WT n=12, AS n=15, p=0.30. **(H)** mRNA levels of *Limk1* in the hippocampus of P21 mice, WT n=12, AS n=15, *p=0.01. **(I)** mRNA levels of *Pumilio2* in the hippocampus of P21 mice, WT n=12, AS n=15, p=0.02. **(J)** Relative expression of miR-134 in the cortex of P21 mice, WT n=10, AS n=10, p=0.14 **(K)** mRNA levels of *Serpine1* in the cortex of P21 mice, WT n=13, AS n=14, p=0.80. **(L)** mRNA levels of Dcx in the cortex of P21 mice, WT n=13, AS n=13, p=0.60. **(M)** mRNA levels of *Limk1* in the cortex of P21 mice, WT n=13, AS n=14, p=0.09. **(N)** mRNA levels of *Pumilio2* in the cortex of P21 mice, WT n=13, AS n=14, **p=0.005. U19 was used as a normaliser for miR-134 levels. β-actin was used as a normaliser for protein coding transcript analysis. A t-test was used for statistical analysis and a Shapiro-Wilk test was used to test for normality. Multiple corrections were performed on molecular data and α was adjusted to 0.0125. Data are expressed as mean ± SEM.

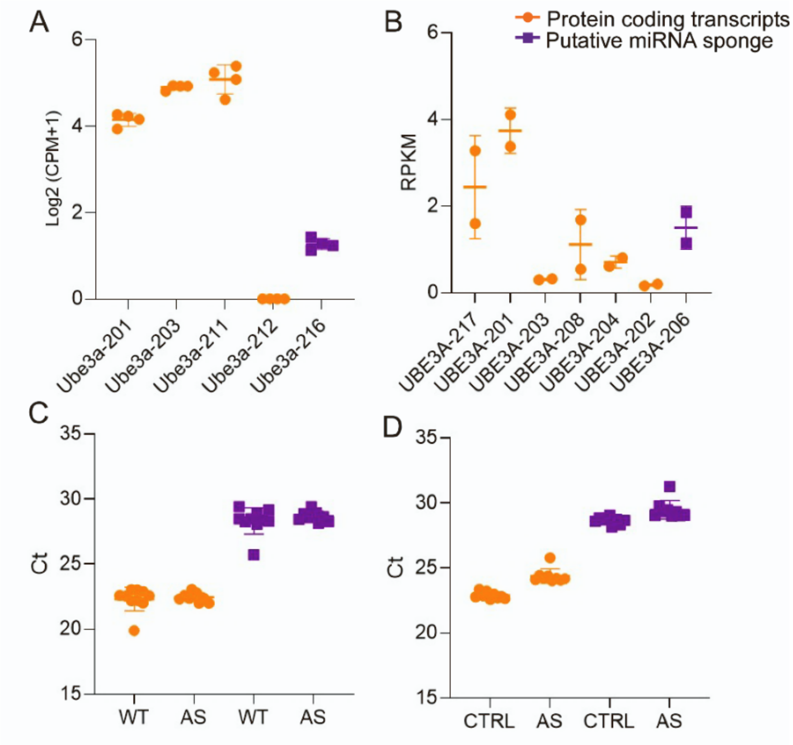

Figure S4: **Expression levels of the Ube3a putative miRNA sponge splicing variant relative to its protein coding variants. (A-B)** Graphs depict representative RNAseq dataset retrieved from the Gene Expression Omnibus (GEO) database for the levels of specific Ube3a/UBE3A splicing variants in the **(A)** hippocampus of WT mice (n=4), and in **(B)** human cortex samples (n=2)**. (C-D)** Graphs show the expression of the selected Ube3a/UBE3A splicing variants measured by RT-qPCR in the **(C)** hippocampi of P24 WT (n=10) and Ube3am$^{-/p+}$ mice (n=9), along with **(D)** control (n=9) and AS (n=9) human iPSCs. Ct= Cycle threshold, CPM=Counts Per Million, RPKM=Reads Per Kilobase per Million.

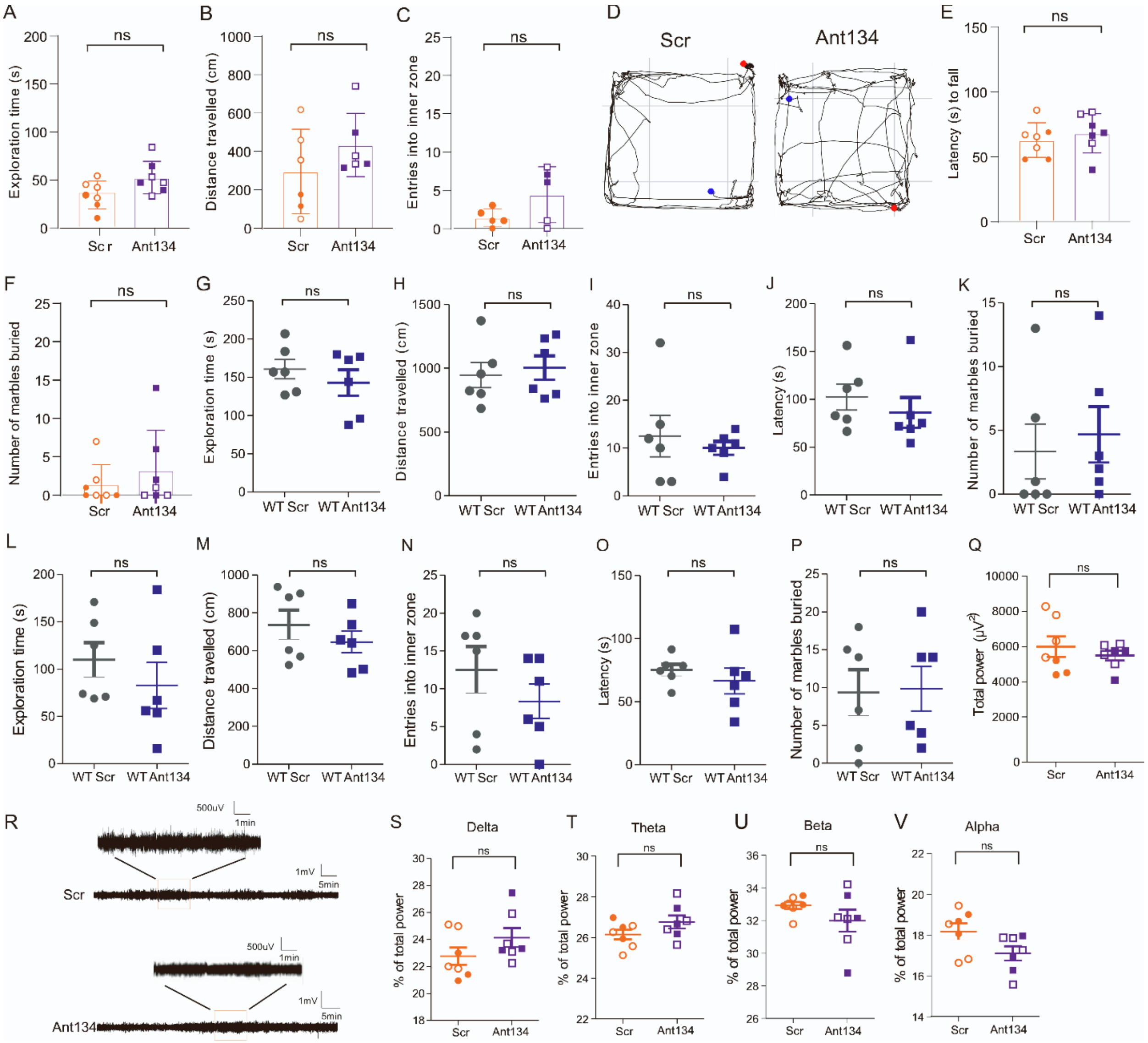

Figure S5: **Re-testing Ant-134-treated F1 AS mice and effects of Ant-134 in wildtype mice. (A-F)** Ant-134/Scr-treated F1 AS mice were re-tested 3 weeks later at P42 and put through the behavioural tests. **(A)** Exploration time (s) during open field in P42 mice, Scr n=7, Ant-134 n=7, p=0.053. **(B)** Distance travelled (cm) in open field arena, Scr n=6, Ant-134, n=6, p=0.245. **(C)** Entries into inner zone of open field arena, Scr n=5, Ant-134 n=5, p=0.117. **(D)** Representative track plot of Scr/Ant-134 mouse at P42 in open field. **(E)** Latency to fall (s) on rotarod, Scr n=7, Ant-134 n=7, p=0.50. **(F)** Number of marbles buried, Scr n=7, Ant-134 n=7, p=0.60. **(G-K)** In a separate experiment, P21 WT mice were given Scr/Ant-134 and 24 h later put through behavioural tests. **(G)** Exploration time (s) during open field, Scr n=6, Ant-134 n=6, p=0.43**. (H)** Distance travelled (cm) in open field arena, Scr n=6, Ant-134 n=6, p=0.68. **(I)** Entries into inner zone of open field arena, Scr n=6, Ant-134 n=6, p=0.60. **(J)** Latency to fall (s) on rotarod, Scr n=6, Ant-134 n=6, p=0.45. **(K)** Number of marbles buried, Scr n=6, Ant-134 n=6, p=0.67. **(L-V)** Re-testing of WT mice at P42 after treatment with Scr/Ant-134 at P21. **(L)** Exploration time (s) during

open field in P42 WT mice, Scr n=6, Ant-134 n=6, p=0.40. **(M)** Distance travelled (cm) in open field arena in P42 WT mice, Scr n=6, Ant-134 n=6. **(N)** Entries into inner zone of open field arena in P42 WT mice, Scr n=6, Ant-134 n=6, p=0.30. **(O)** Latency to fall (s) on rotarod in P42 WT mice, Scr n=6, Ant-134 n=6, p=0.45. **(P)** Number of marble buried in P42 WT mice, Scr n=6, Ant-134 n=6, p=0.91. The effect of Ant-134 on baseline EEG and behavioural phenotypes in F1 mice was analysed. P42 mice were pre-treated with Scr/Ant-134 and resting EEG was recorded 24 h later. **(Q)** Total power (μV^2) of baseline EEG in Scr and Ant-134 mice, n=7/group, p=0.45. **(R)** Representative EEG traces for Scr and Ant-134 mice of 1 h recordings, scale bar 1 mV, 5 min. Zoom scale bar shows 10 minute recording, 500 uV, 1 min. **(S)** % of total power of delta, n=7/group, p=0.17. **(T)** % of total power of theta, n=7/group, p=0.14. **(U)** % of total power of beta, n=7/group, p=0.21. **(V)** % of total power of alpha, n=7/group, p=0.06.

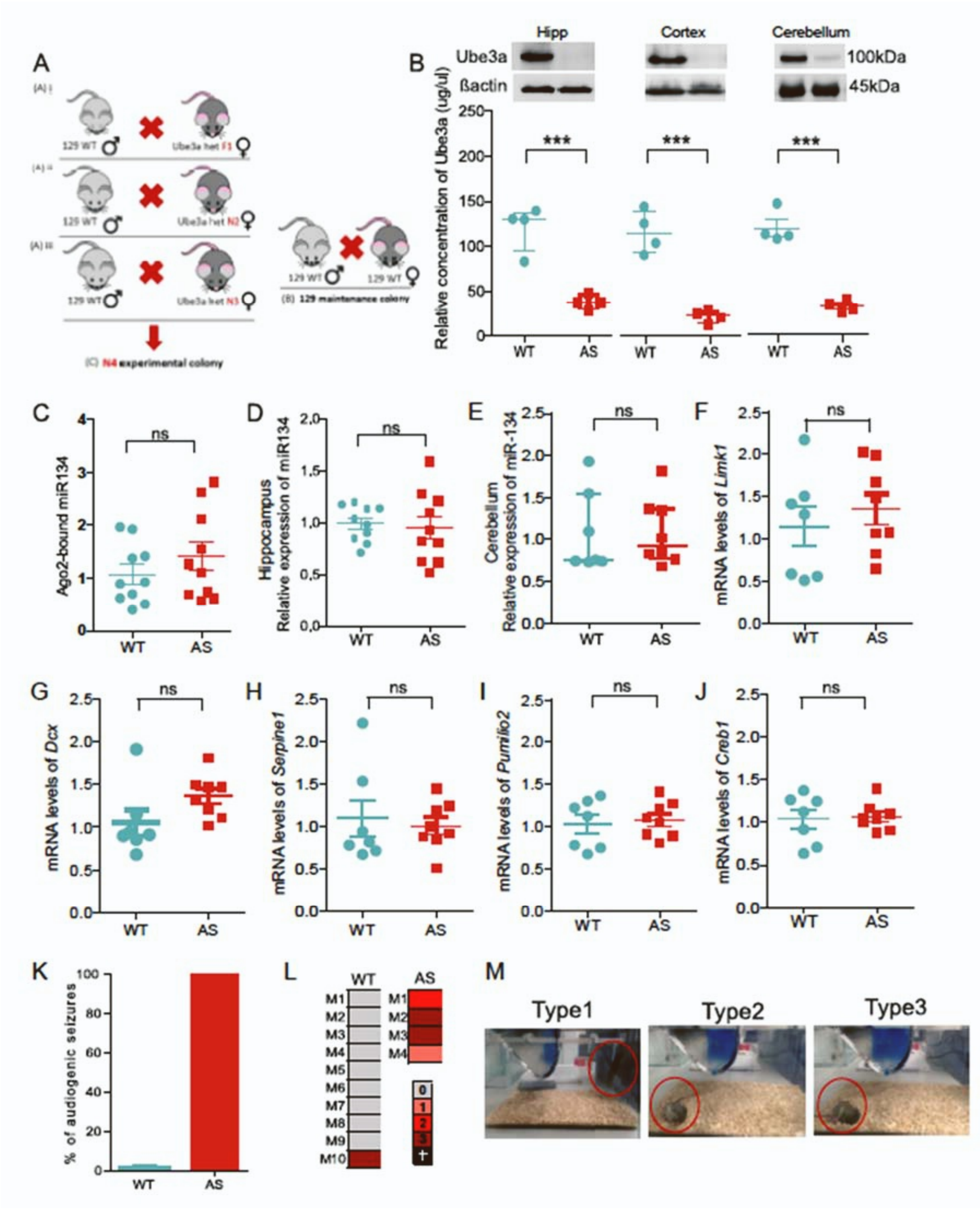

Figure S6: Generation of AS mice susceptible to audiogenic seizures. Generation of N4 colony AS mice susceptible to audiogenic seizures. **(A)** Breeding scheme of N4 colony by backcrossing from generation F1 *(Ai)* to generate N3 female mice *(Aiii)*. N3 female mice were crossed with the 129 maintenance colony *(Aii)* to yield the N4 experimental colony *(Aiv)*. **(B&C)** Western blot showing protein levels of Ube3a in the hippocampus, cortex and cerebellum of P21 N4 mice. Semi-quantification of Ube3a levels in the hippocampus (WT n=4, AS n=4, ***p=0.0008),

cortex (WT n=4, AS n=4 ***p=0.0003) and cerebellum (n=4 WT, AS n=4, ***p=0.0286). **(C)** Relative expression of Ago-2 bound miR-134 in the hippocampus at P21, WT n=10, AS n=10, p=0.312. **(D)** Relative expression of miR-134 in the hippocampus of N4 P21 mice, WT n=10, AS n=10 p= 0.723. **(E)** Relative expression of miR-134 in the cerebellum of N4 P21 mice, WT n=7, AS n=8, p=0.693. **(F)** mRNA levels of *limk1* in the cerebellum of N4 P21 mice,WT n=7, AS n=8, p=0.5139. **(G)** mRNA levels of *Dcx* in the cerebellum of N4 P21 mice, WT n=7, AS n=8, p=0.09. **(H)** mRNA levels of *Serpine1* in the cerebellum of N4 P21 mice, WT n=7, AS n=8, p=0.693. **(I)** mRNA levels of *Pumilio2* in the cerebellum of N4 P21 mice, WT n=7, AS n=8, p=0.709. **(J)** mRNA levels of *Creb1* in the cerebellum of N4 P21 mice, WT n=7, AS n=8, p=0.796. **(K)** N4 P21 AS mice are susceptible to audiogenic-induced seizures (WT n=10, AS n=4). **(L)** Individual seizure characterization using a scoring scale for audiogenic seizures. Type 0: No seizure activity, Type 1: Wild running when stimulus is initiated, Type 2: Generalized tonic-clonic convulsions with loss of posture, Type 3: Tonic hyperextension of the hindlimbs and tail and Type 4: Death. **(M)** Screenshots from videos recording elicited audiogenic seizures and covering examples of types 1-3. Data are expressed as SEM or median and interquartile range. A *t*-test or a Mann-Whitney test was used for analysis between WT and AS mice.

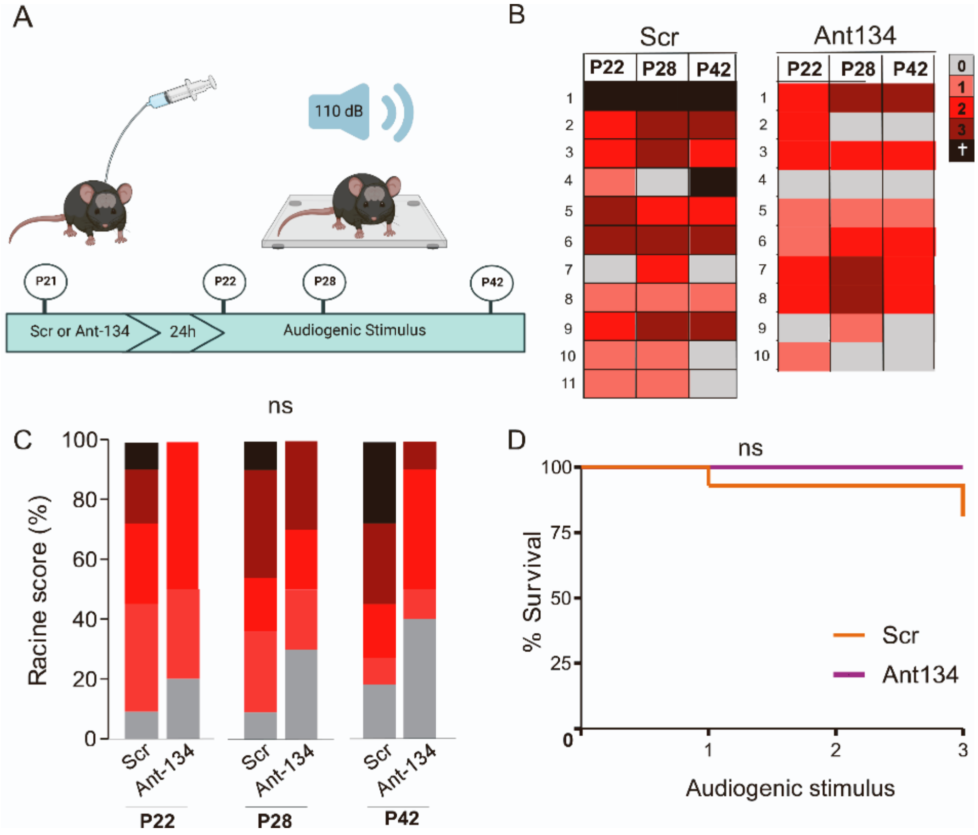

Figure S7: Assessment of longer-term effects of Ant-134 on audiogenic-induced seizures. (A) N4 P21 AS mice were pre-treated with 0.5 nmol/2µl of Ant-134 or Scr (ICV) and subjected to three individual audiogenic stimuli at P22, then at P28 and finally at P42. Image drawn using Biorender. **(B)** Tabular representation of seizure severity per mouse per time point following Scr/Ant-134 (0.5 nmol/2µl; i.c.v.; Scr n=11, Ant-134 n=10). **(C)** Scale representing seizure severity (% per type 0-4) over the course of three time points (P22, P28 and P42; Scr n=11, Ant-134 n=10, p=0.22). **(D)** Percentage of survival of mice pre-treated with Scr/Ant-134 (0.5 nmol) over the course of three seizures, Scr n=11, Ant-134 n=10. Data were analysed with Stata Release 16.1. Mortality was compared between treatments using incidence rate ratios and a Poisson model, with number of trials as the exposure variable. Seizure severity was modelled using ordinal logistic regression with robust variance estimation used to adjust for clustering of data with mice. An interaction term was used to test for a change in effectiveness of treatment as a function of trial number. For the survival curve analysis, a Log-rank (Mantel-Cox) test was used.

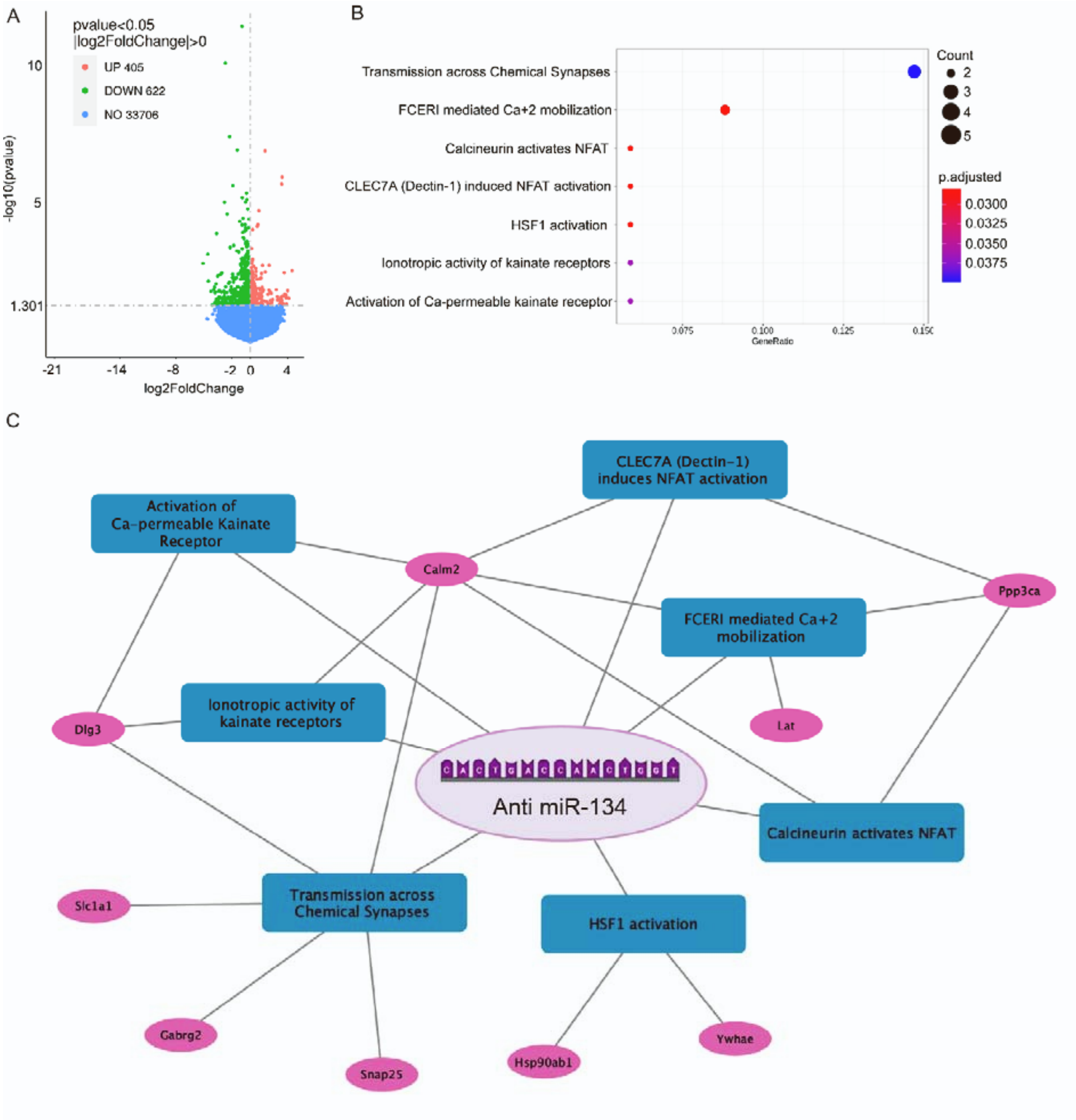

Figure S8: Gene expression profile after Ant-134 treatment in AS mice. (A) Volcano plot of the Differentially Expressed Genes (DEGs) between Ant-134 (n=5) and Scr-treated (n=4) AS mice identified using RNAseq. Genes with a p-value <0.05 and a |Log2 Fold Change| > 0 were considered differentially expressed. 405 genes were upregulated and 622 genes were downregulated in the Ant-134 mice. **(B)** Reactome Pathways enriched among DEGs of interest. Reactome pathway enrichment was performed on the list of DEGs of interest. Pathways with an adjusted p-value <0.05 were considered significantly enriched. The Gene Ratio is the number of DEGs of interest annotated to a given pathway over the total genes annotated to the same pathway. The Background Ratio is the number of total genes annotated to a given pathway over the total number of genes represented in the Reactome database. **(C)** Reactome Pathways enriched among DEGs of interest. Network of the significantly enriched Reactome Pathways and their associated DEGs of interest.

Supplementary movie titles and legends

SI Movie M1

Representative video of a P22 AS mouse in the open field that had been treated 24 h earlier with scrambled.

SI Movie M2

Representative video of a P22 AS mouse in the open field that had been treated 24 h earlier with Ant-134.

SI Movie M3

Representative video of a P22 AS mouse response to audiogenic seizure stimulus that had been treated 24 h earlier with scrambled.

SI Movie M4

Representative video of a P22 AS mouse response to audiogenic seizure stimulus that had been treated 24 h earlier with Ant-134.

SI Movie M5

Movie showing spontaneous network activity in Fluo-4 loaded neurons differentiated from Angelman iPSC line. The fluorescence variation is shown in pseudo-colours. Scale bar: 30 µm.

Reference

28. Reschke, C.R., Silva, L.F.A., Vangoor, V.R., Rosso, M., David, B., Cavanagh, B.L., Connolly, N.M.C., Brennan, G.P., Sanz-Rodriguez, A., Mooney, C. et al. (2021). Systemic delivery of antagomirs during blood-brain barrier disruption is disease-modifying in experimental epilepsy. Mol. Thera. *29*, 2041-2052.
